# Fabrication of Gold and Silver Nanoparticles Supported on Zinc Imidazolate Metal–Organic Frameworks as Active Catalysts for Hydrogen Release from Ammonia Borane

**DOI:** 10.1021/acsomega.4c07068

**Published:** 2024-09-18

**Authors:** Elibe S. Souza, Maria Alaide de Oliveira, Jildimara de Jesus Santana, Natalia Łukasik, Ohanna Maria Menezes Madeiro da Costa, Luciano Costa Almeida, Bráulio Silva Barros, Joanna Kulesza

**Affiliations:** † Programa de Pós-Graduação em Ciência de Materiais, Centro de Ciências Exatas e da Natureza-CCEN, Universidade Federal de Pernambuco, Cidade Universitária, Avenida Jornalista Aníbal Fernandes, s/n°, Recife, Pernambuco 50740-560, Brazil; ‡ Programa de Pós-Graduação em Química, Centro de Ciências Exatas e da Natureza-CCEN, Universidade Federal de Pernambuco, Cidade Universitária, Avenida Jornalista Aníbal Fernandes, s/n°, Recife, Pernambuco 50740-560, Brazil; § Brazilian Synchrotron Light Laboratory (LNLS), Brazilian Center for Research in Energy and Materials (CNPEM), Cidade Universitária, Rua Giuseppe Máximo Scolfaro, Campinas, São Paulo 13083-100, Brazil; ∥ Departamento de Engenharia Química, Centro de Tecnologia e Geociências - CTG, Universidade Federal de Pernambuco, Cidade Universitária, Rua Artur de Sá, Recife, Pernambuco 50740-521, Brazil; ⊥ Departamento de Engenharia Mecânica, Centro de Tecnologia e Geociências - CTG, Universidade Federal de Pernambuco, Cidade Universitária, Av. Prof. Morais Rego, 1235, Recife, Pernambuco 50670-901, Brazil; # Departamento de Química Fundamental, Centro de Ciências Exatas e da Natureza-CCEN, Universidade Federal de Pernambuco, Cidade Universitária, Av. Prof. Morais Rego, 1235, Recife, Pernambuco 50670-901, Brazil

## Abstract

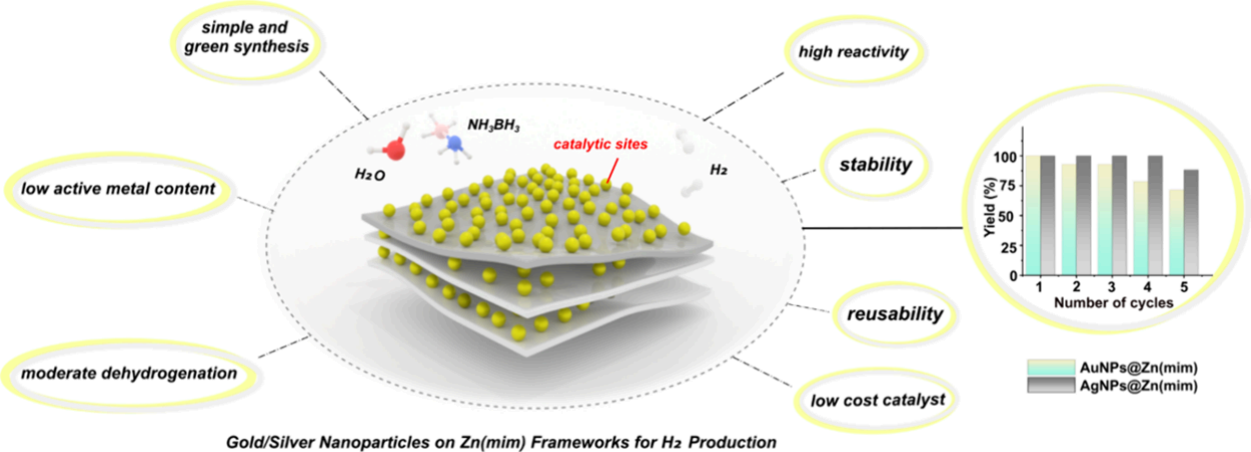

Well-dispersed Au and Ag nanoparticles (NPs) have been immobilized on a zinc imidazolate metal–organic framework, Zn(mim), using the “one-pot” method and tested as catalysts in ammonia borane hydrolysis. The AuNPs@Zn(mim) and AgNPs@Zn(mim) materials were characterized by FTIR, XRD, ICP-OES, TGA, BET, SEM, and TEM. The AgNPs@Zn(mim) catalyst showed a high yield (98.5%) and high hydrogen generation rate (3352.71 mL min^–1^ g_Ag_^–1^) in NH_3_BH_3_ dehydrogenation. The determined activation energies (19.6 kJ mol^–1^ for AuNPs@Zn(mim) and 38.13 kJ mol^–1^ for AgNPs@Zn(mim)) are lower than those for most reported catalysts containing Au/Ag-MOF used in the hydrolysis of NH_3_BH_3_. Moreover, the catalysts tested here revealed good stability and reusability, preserving 71.42% (AuNPs@Zn(mim)) and 88.23% (AgNPs@Zn(mim)) of their initial catalytic activities after five consecutive cycles. In the case of AgNPs@Zn(mim), the combination of the simple and green synthesis method, low active metal content, relatively low cost, and moderate dehydrogenation conditions makes the material an excellent candidate to produce hydrogen from ammonia borane.

## 1 Introduction

Hydrogen is considered to be the fuel of the future. It is a promising, clean, and renewable alternative to meet the emerging need to replace CO_2_-emitting fossil fuels.^1^ Molecular hydrogen (H_2_) has greater energy per mass content than gasoline (143 MJ kg^–1^ vs 46.4 MJ kg^–1^).^2^ Upon combustion, only water is generated, making it a promising option to mitigate climate change. However, storage of H_2_ is particularly problematic as it is a low-density gas at ambient conditions, resulting in a low energy per volume value (12.7 MJ m^–3^ vs 40 MJ m^–3^ for CH_4_);^1^ thus, relatively large-volume pressure tanks are required. On the other hand, storing H_2_ in the liquid form is difficult because a very low temperature (−253 °C) is needed to liquefy the hydrogen.^3^ In addition, the current lack of logistics, transport infrastructure, and distribution of molecular hydrogen to final consumers requires high investments.^4^ Consequently, new and advanced storage methods that allow higher volumetric energy densities have been explored in the last two decades. There are two types of hydrogen storage: physical and chemical.^1^ Chemical hydrogen^5^ storage can be considered one of the most promising approaches for safely and efficiently storing hydrogen.^6^ In this case, hydrogen is obtained by dehydrogenating H_2_-rich molecules under moderate conditions (depending on the type of H_2_ carrier). Since molecular hydrogen burns in the presence of air, handling solid or liquid compounds with covalently bonded hydrogen is safer and easier.^4^ The hydrogen produced on demand can be used in fuel cells,^7−10^ which convert chemical energy into electricity and heat within an electrochemical reaction in which water is the only reaction product. However, the state of the art in using hydrogen energy conversion technologies through fuel cells needs improvements in the feasibility/cost ratio.^4^ The simplest H_2_ carriers are alcohols such as methanol or ethanol,^11^ formic acid (HCOOH),^12,13^ formaldehyde (HCHO),^14^ sodium borohydride (NaBH_4_),^15^ ammonia borane (NH_3_BH_3_),^16^ and borane hydrazine (N_2_H_4_BH_3_).^17^

NH_3_BH_3_ is a promising molecule in the production and storage of chemical hydrogen because of its high hydrogen capacity (19.6 wt %), low molecular weight (30.9 g mol^–1^), high stability in the solid state and aqueous solution under ambient conditions, no toxicity, and high solubility, which allow its easy handling at ambient temperature and pressure.^10^ However, NH_3_BH_3_ solution is highly resistant to hydrolysis under ambient conditions, requiring an active and stable catalyst to accelerate the reaction.^18,19^ Upon NH_3_BH_3_ hydrolysis, the interaction between the NH_3_BH_3_ molecule and the catalyst surface leads to some active complexes that dissociate to the B–N bonds after attack by the H_2_O molecule. Hydrolysis of the resulting BH_3_ intermediate yields the boric acid product and the release of H_2_, using a hydrogen atom from the -BH_3_ group of NH_3_BH_3_ and one from a water molecule (eq 1).^18^
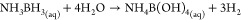
1Therefore, the accessibility of the active sites of the catalyst is the key factor determining the effectiveness of the hydrolysis reaction. Metallic nanoparticles (MNPs) have proven high activity in dehydrogenation processes; however, they are thermodynamically unstable and consequently tend to agglomerate, decreasing the exposure of active sites and weakening catalytic properties.^10,20,21^ Metal–organic frameworks (MOFs) are promising candidates for heterogeneous catalyst supports for the conversion of molecules to hydrogen due to their high porosity, chemical stability, and tunable structure.^22−25^

The incorporation and dispersibility of MNPs in MOFs allow the development of catalysts with more stability, high surface area, porosity, better adsorption, and diffusion of substrates and products by mass transport. Some catalysts like Pd-zeolite,^26^ Pd@MIL-101,^27^ AgPd@UIO-66-NH_2,_^28^ AuCo@MIL-101,^29^ AuNi@ZIF-8,^30^ Ru-MIL-53 (Cr, Al),^31^ Ni/ZIF-8,^10^ and Ag@Zn-MOF^32^ containing MNPs as an active part and MOF as the support have been used in the dehydrogenation reaction of NH_3_BH_3_. However, one of the main limitations of using these catalysts is the high cost of production since the most active materials were synthesized with costly precious metals.

This work presents Zn-imidazolate-based catalysts as a porous support for gold and silver nanoparticles obtained via environmentally friendly synthesis (in water). Although precious metals form catalyst active sites, the low content in the resulting materials positively influences the catalyst price, ensuring high catalytic activity. To the best of our knowledge, the materials have not been reported to date. Here, we describe the successful synthesis and characterization of AgNPs@Zn(mim) and AuNPs@Zn(mim) catalysts and their application in hydrogen generation via NH_3_BH_3_ hydrolysis.

## 2 Materials and Methods

All reagents and solvents were used without further purification. Silver nitrate (AgNO_3_) (99%), NH_3_BH_3_ (97%), NaBH_4_ (98%), 2-methylimidazole (Hmim) (C_4_H_6_N_2_) (99%), and chloroauric acid trihydrate (HAuCl_4_ × 3H_2_O) (99.9%) were purchased from Sigma-Aldrich. Zinc acetate dihydrate (C_4_H_6_O_4_Zn × 2H_2_O) (PA) and ammonium hydroxide (NH_4_OH) (PA) were purchased from Vetec and Moderna, respectively. Copper sulfate (CuSO_4_·5H_2_O) (PA) and sodium hydroxide (NaOH) (PA) were purchased from Dinamica.

### 2.1 Synthesis of Zn(mim) Support

The synthesis of Zn(mim) support was based on a procedure described in the literature.^33^ In a beaker, 586 mg (2.8 mmol) of zinc acetate and 1.16 g (23.3 mmol) of the organic ligand 2-methylimidazole (Hmim) were dissolved in 60 mL of distilled water and left under vigorous stirring for 24 h at 27 °C. After obtaining a white powder, the product was centrifuged, washed with distilled water, and dried at 60 °C in an oven for 18 h.

### 2.2 Incorporation of AuNPs and AgNPs in Zn(mim) Support by the One-Pot Method

In a beaker, 586 mg (2.8 mmol) of zinc acetate, 1.16 g (23.3 mmol) of the organic ligand 2-methylimidazole (Hmim), and 40 mg of an MNP precursor (AgNO_3_ or HAuCl_4_) were dissolved in distilled water and left under vigorous stirring for 24 h at 27 °C. The material (M^*n*+^@Zn(mim), M = Au or Ag) was centrifuged, and the metal ions were then reduced to MNPs according to the following procedure.^34,35^ The obtained material (M^*n*+^@Zn(mim), M = Au or Ag) was dispersed in 15 mL (sample with Au) and 160 mL (sample with Ag) of distilled water for 15 min using a magnetic stirrer in the presence of an ice bath. Next, an aqueous solution of NaBH_4_ (2 mmol L^–1^) was slowly dropped (1 drop/second) into the solution of Au^3+^@Zn(mim) and Ag^+^@Zn(mim) (volume of added NaBH_4_: 23 and 15 mL, respectively). With the addition of the reducing agent, a color change was observed: from yellow to pinkish in the gold sample, resulting from the reduction of Au^3+^@Zn(mim) to AuNPs@Zn(mim), and from white to yellow in the silver sample, resulting from the reduction of Ag^+^@Zn(mim) to AgNPs@Zn(mim). These colors remained in the powder materials. After forming MNPs, the resulting material was left under stirring in an ice bath for 20 min. The obtained product was washed with water, centrifuged, and dried at 60 °C in an oven for 18 h. The synthesis of the materials was performed at least three times.

### 2.3 Characterization

Spectra in the UV–visible region were acquired using a UV–vis spectrophotometer model UV-M51 from BEL Engineering. The Fourier transform infrared (FTIR) spectra were acquired on Bruker FTIR IFS66 equipment from 400 to 4000 cm^–1^ by a transmission technique (KBr pellets). Infrared measurements were also performed on the Imbuia beamline at the Brazilian Synchrotron Light Laboratory - Sirius using the Agilent Cary 670 spectrometer. These spectra were collected in the 400–4000 cm^–1^ range with a resolution of 8 cm^–1^ by the KBr wafer transmission mode. All presented micro-FTIR transmittance spectra corresponded to the average of 128 single spectra. The powder X-ray diffraction (XRD) patterns of the prepared samples were recorded on a Shimadzu XRD 7000 diffractometer using CuKα radiation (λ = 1.5418 Å), with a step of 0.02° (2θ = 5° to 80°). The identification of phases ZIF-8 and dia(Zn) was based on files 4118891 Crystallography Open Database - COD and 783838 Cambridge Crystallographic Data Center - CCDC, respectively. The crystallite sizes of the phases of each sample were calculated using the Scherrer equation (see the Supporting Information), which includes the instrumental correction based on the three most intense peaks of the PXRD patterns. The percentages of the crystalline phases were identified by calculating the ratio of the integrated area of all crystalline peaks to the total integrated area under the XRD peaks, as calculated from the patterns of each phase.

The simulated diffraction patterns were obtained by using the Mercury 3.8 program. Inductively coupled plasma-optic emission spectroscopy (ICP-OES) analysis was performed to investigate the mass content of Au and Ag metals in the catalysts using the Thermo Fisher Scientific spectrometer (Bremen, Germany), model iCAP 6300 Duo, with an axial and radial view and a simultaneous detector charge injection device. The power of the radiofrequency source was 1150 W, the gas flow rate was 0.5 L min^–1^, and each sample was analyzed in triplicate. The samples were digested in nitric acid (40 mL), and the wavelengths used were 242.8 nm (Au) and 338.3 nm (Ag). The textural properties of catalysts (BET surface area, specific volume, and pore size) were obtained from the adsorption–desorption of nitrogen at 77.3 K using the Quantachrome Autosorb-iQ Instruments. Before the measurements, the samples were degassed at 80 °C for 24 h in a vacuum oven. Thermogravimetric analysis (TGA) was conducted on a Shimadzu DTG-60H apparatus. The samples were heated in the 23–800 °C range at a heating rate of 10 °C min^–1^ under a nitrogen atmosphere. The morphology of the samples was examined using scanning electron microscopy (SEM) in high-resolution MIRA 3 Tescan equipment with an accelerating voltage of 10 kV. A scanning electron microscope with an energy-dispersive X-ray spectroscopy (EDS) detector was also used for elemental mapping in Helios 3 PFIB CXE Dual Beam equipment. Particle size histograms were constructed by counting at least 100 particles by using ImageJ software. Transmission electron microscopy (TEM) images were acquired on an FEI Tecnai G2 Spirit Biotwin with a resolution of 0.34 nm, voltage of 120 kV, tungsten filament (W), and magnification up to 300.000×.

### 2.4 Catalytic Activity Tests

The catalytic activity in the hydrolysis of NH_3_BH_3_ of the synthesized catalysts was evaluated using an experimental setup proposed by Zanchetta et al.^36^ The water displacement method allowed us to determine the volume of hydrogen gas generated from the hydrogen storage molecule. The experimental system consists of two main parts. (I) The first is a temperature-controlled water bath reactor with magnetic stirring where the catalyst and reagents are added. The hydrolysis reaction is initiated by adding the NH_3_BH_3_ solution to the reactor, followed by the addition of the catalyst. (II) The second is a container with an inverted test tube submerged in water that receives the gas produced through a hose connected to the condenser attached to the flask. The amount of H_2_ produced is measured by the displacement of the volume of water caused by the generated gas.

The experiments were performed at 40, 50, 60, and 70 ± 0.2 °C using 50 mg of a catalyst and 5 mL of 0.13 mol L^–1^ NH_3_BH_3_. All experiments were performed in triplicate and monitored for an average time of 60 min. Control experiments were carried out to verify if the bare nanoparticles (AuNPs, 1 wt %, and AgNPs, 1 wt %) and the used support (Zn(mim)) exhibit some catalytic activity in the hydrolysis of NH_3_BH_3_ (for the synthesis of AuNPs and AgNPs, see the supporting material). Also, NH_3_BH_3_ autohydrolysis was tested. These studies were performed by using 5 mL of 0.13 mol L^–1^ NH_3_BH_3_ at 40 ± 0.2 °C.

The effect of NaOH presence on hydrogen generation was investigated by adding different concentrations of NaOH (1, 2, 3, and 4 mol L^–1^) to the reaction medium containing 50 mg of AgNPs@ Zn(mim) catalyst and 5 mL of 0.13 mol L^–1^ NH_3_BH_3_ at 40 ± 0.2 °C. The equations for parameter calculations (turnover frequency, TOF; hydrogen generation rate, HGR; activation energy, Ea) that evaluate the performance of the catalyst are shown in the supporting material.

To ensure that no ammonia is released together with H_2_, the following experiment was conducted. An aqueous NH_3_BH_3_ solution (5 mL, 0.398 wt %) was introduced into a two-neck round-bottom flask equipped with a septum inlet and a reflux condenser. This flask was connected to a sealed flask containing a CuSO_4_ solution (100 mL, 0.5 mol L^–1^) through a hose. The AgNPs@Zn(mim) catalyst (0.76 mol %) was then added to the flask, and the resulting gas was collected over 60 min, utilizing the highest temperature employed in the studies, 70 °C. A reference experiment was conducted without a catalyst, using NH_4_OH (5 mL) instead of NH_3_BH_3_ as the NH_3_ precursor at 70 °C.

### 2.5 Reuse of Catalysts

For the reuse tests, the first cycle was performed as described above for the hydrolysis of NH_3_BH_3_ with 50 mg of the catalyst (AuNPs@Zn(mim) or AgNPs@Zn(mim)) and 5 mL of 0.13 mol L^–1^ NH_3_BH_3_ at a temperature of 40 ± 0.2 °C. After almost 1 h of the reaction, the solution was centrifugated (10 min, 3000 rpm), and the catalysts recovered without further purification. Then, a fresh portion of the NH_3_BH_3_ solution was added to the reactor to carry out the next cycle. This procedure was performed in five consecutive cycles of NH_3_BH_3_ hydrolysis.

## 3 Results and Discussion

### 3.1 Catalyst Characterization

The synthesized samples of Zn(mim) had a white color, and the prepared composites, AuNPs@Zn(mim) and AgNPs@Zn(mim), presented lilac and yellow colors, respectively, characteristic of each metal in nanometric size according to the literature (Figure S1).^37,38^ The consistency in the diffractograms in Figure S2 shows the reproducibility of the material synthesis in different repetitions, indicating that the experimental conditions were efficiently controlled, contributing to the reliability of the results.

#### 3.1.1 μ-FTIR

**Figure 1:**
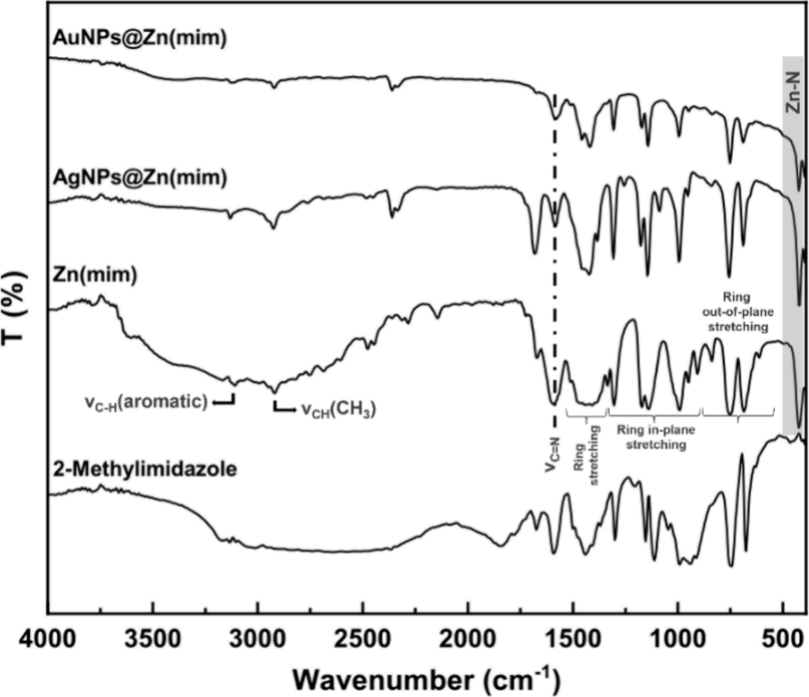
μ-FTIR spectra of the prepared samples, Zn(mim) and MNPs@Zn(mim), compared to the spectrum of the ligand, 2-methylimidazole.

Infrared measurements with a spectral resolution of 8 cm^–1^ were made to structurally characterize the samples of Zn(mim) and nanocomposites MNPs@Zn(mim) (Figure 1). In all registered spectra, bands characteristic of ZIF-8 were observed.^39−44^

Most of the absorption bands observed in the Zn(mim) spectrum are associated with the vibrations of the imidazole ring. The bands at 3110 and 2921 cm^–1^ are attributed to the aromatic and aliphatic C–H stretching of imidazole molecules, respectively. The bands at 1679 and 1585 cm^–1^ correspond to the vibrations of the imidazole ring skeleton; however, there are some intensity changes in the infrared spectra of the nanocomposites, mainly in AgNPs@Zn(mim). This result indicates that interactions between NPs and the ligand exist.^45^

The spectra of the nanocomposites are consistent with that of Zn(mim), suggesting the successful loading of Ag and Au without destroying the structure of Zn(mim), indicating its high stability.^46^ When comparing the Zn(mim) spectrum with those of nanocomposites, it is observed that the nanocomposites present a decrease in the relative intensity of some bands, indicating the presence of interaction signals among AgNPs, AuNPs, and Zn(mim).^47^ Finally, the bands at 600–1500 cm^–1^ are associated with elongation or flexion of the entire ring,^39^ and the band located at 428 cm^–1^ is attributed to the Zn–N stretching vibration, which indicates the coordination between Zn^2+^ and 2-methylimidazole.^39,48^

#### 3.1.2 XRD

**Figure 2:**
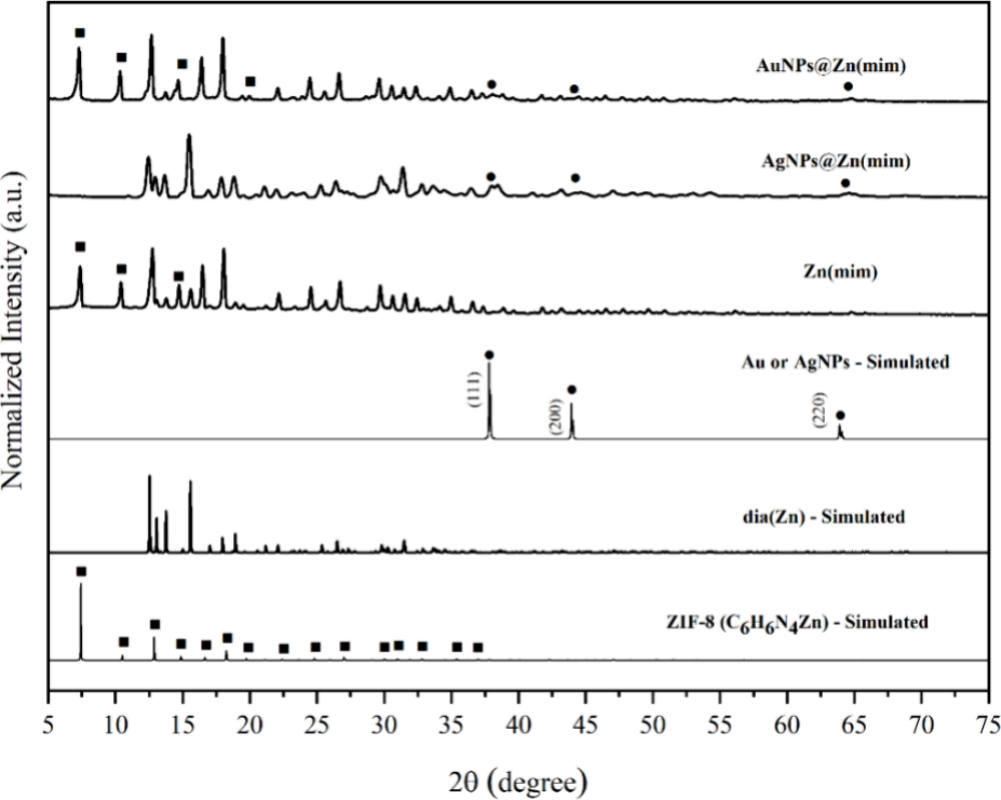
Diffractograms of Zn(mim) and MNPs@Zn(mim) samples compared to the simulated patterns of ZIF-8, dia(Zn), and Au/Ag nanoparticles.

The XRD patterns of Zn(mim) and composites (MNPs@Zn(mim)) compared to the simulated patterns are shown in Figure 2.

The samples showed crystalline structures after incorporation of MNPs. Despite the low amount of metal, the presence of diffraction peaks characteristic of MNPs was observed, consistent with the simulated patterns for gold and silver (COD numbers 9013040 and 1100136, respectively).

Two different crystalline phases were identified from the XRD patterns of the samples; however, incorporating Ag nanoparticles into the Zn(mim) support led to almost pure-phase formation. One of the crystalline phases is ZIF-8,^44^ which is observed for samples Zn(mim), AuNPs@Zn(mim), and AgNPs@Zn(mim) in amounts of about 65.1, 70.3, and 1.8%, respectively. The second phase, cited by some authors as one of the polymorphs of ZIF-8 resulting from the hydrolysis (dissolution) of ZIF-8,^49,50^ is observed in XRD patterns of Zn(mim), AuNPs@Zn(mim), and predominantly in the AgNPs@Zn(mim) pattern with 34.9, 29.7, and 98.2%, respectively.^34,50^ The two structures, ZIF-8 and dia (Zn), are shown in Figure S3. The polymorph is known as dia(Zn): Zn(mim)_2_, mim = 2-methylimidazolate, and is formed from zinc acetate Zn(OAc)_2_ and the excess of the organic ligand. In this structure, each Zn^2+^ ion is coordinated by four N atoms of the bridged 2-methylimidazolate groups.^50^

The crystallite sizes of the synthesized materials presented similar values (Table 1). The exception is the AgNPs@Zn(mim) sample with the predominant dia(Zn) crystalline phase, for which a smaller crystallite size value was found.

**Table 1:** Crystallite Sizes of the Zn(mim) Samples^a^

sample	crystallite size (nm) phase dia (Zn)	crystallite size (nm) phase ZIF-8
Zn(mim)	104.81	92.68
AuNPs@ Zn(mim)	104.81	104.30
AgNPs@ Zn(mim)	48.88	nd

a nd, not possible to determine.

#### 3.1.3 ICP-OES

The quantification of MNPs in the catalysts was determined by ICP-OES. The results of the ICP-OES analysis showed 0.89 wt % Au and 1.06 wt % Ag for samples AuNPs@Zn(mim) and AgNPs@Zn(mim), respectively. The small quantity of MNPs corroborates the low intensity of diffraction peaks attributed to the metallic nanocrystals.

#### 3.1.4 TGA

**Figure 3:**
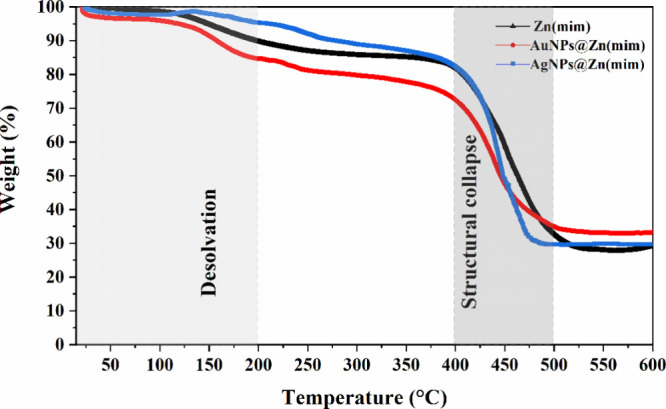
TGA curves of samples Zn(mim), AgNPs@Zn(mim), and AuNPs@Zn(mim).

The thermogravimetric curves of the synthesized samples are shown in Figure 3.

The thermograms show that the Zn(mim), AgNPs@Zn(mim), and AuNPs@Zn(mim) samples presented the first thermal event below 200 °C, related to the loss of adsorbed water with a gradual loss of 12.7, 9.5, and 19%, respectively. Then, a plateau was observed after the formation of the Zn(mim) guest-free phase up to approximately 400 °C, indicating the good thermal stability of the 3D network for all synthesized samples. Most of the significant weight loss of these nanocrystals (47.8–60.2%) occurred upon increasing the temperature from 400 to 500 °C. The polymorphism of the crystalline phases (ZIF-8 and dia(Zn)) justifies the similarity of the thermal decomposition profile of these crystals.^49^ These results are in agreement with the literature.^34,49^

#### 3.1.5 BET

The N_2_ adsorption–desorption isotherms are shown in Figure S4. According to the IUPAC classification, the isotherms are presented as type II, typical for nonporous materials.^46^ However, the pore sizes are below 35 nm and can be classified as mesopores (Figure S4D). A small hysteresis (the resulting isotherm does not follow the same path for desorption) was observed at low pressures, with greater difficulty in desorbing the N_2_ molecules condensed in the smaller pores (Figure S4A–C). According to the literature, N_2_ removal is possible only if the adsorbent is degassed at higher temperatures. This phenomenon may be associated with the expansion of a nonrigid porous structure or the irreversible absorption of molecules in pores of approximately the same width as the N_2_ molecule or, in some cases, with an irreversible chemical interaction of the adsorbate with the adsorbent.^51^ In our case, the temperature (80 °C) is likely insufficient to remove all of the N_2_ from the pores.

The BET results (Table 2) of the nanocomposites showed a considerable decrease (785.01 m^2^ g^–1^ for AuNPs@Zn(mim) and 25.26 m^2^ g^–1^ for AgNPs@Zn(mim)) in relation to the support Zn(mim) (916.59 m^2^ g^–1^). Such a drop is related to the crystalline phase present in the samples, so that the greater the quantity of the dia(Zn) phase in the material, the smaller the surface area (BET) and pore volume since dia(Zn) is a polymorph of ZIF-8, in which zinc(II) and 2-methylimidazole are more densely packed.^49^ This is consistent with the literature data, where a reduction from 1291 m^2^ g^–1^–0.681 cc g^–1^ (ZIF-8) to 11.4 m^2^g^–1^– 0.000611 cc g^–1^ (polymorph dia (Zn)) was observed.^49,50^ Furthermore, the encapsulation of MNPs in the cavities or the blocking of pores of the metal–organic structure by MNPs on the surface might also be responsible for the small surface area and pore volume, especially for the AgNPs@Zn(mim) sample.^26,49,50,52^

**Table 2:** Texture Properties of Samples Zn(mim) and MNPs@Zn(mim)

sample	surface area (BET) (m^2^ g^–1^)	total pore volume (cc g^–1^)
Zn(mim)	916.59	0.5649
AuNPs@Zn(mim)	785.01	0.4557
AgNPs@Zn(mim)	25.26	0.07928

#### 3.1.6 SEM

**Figure 4:**
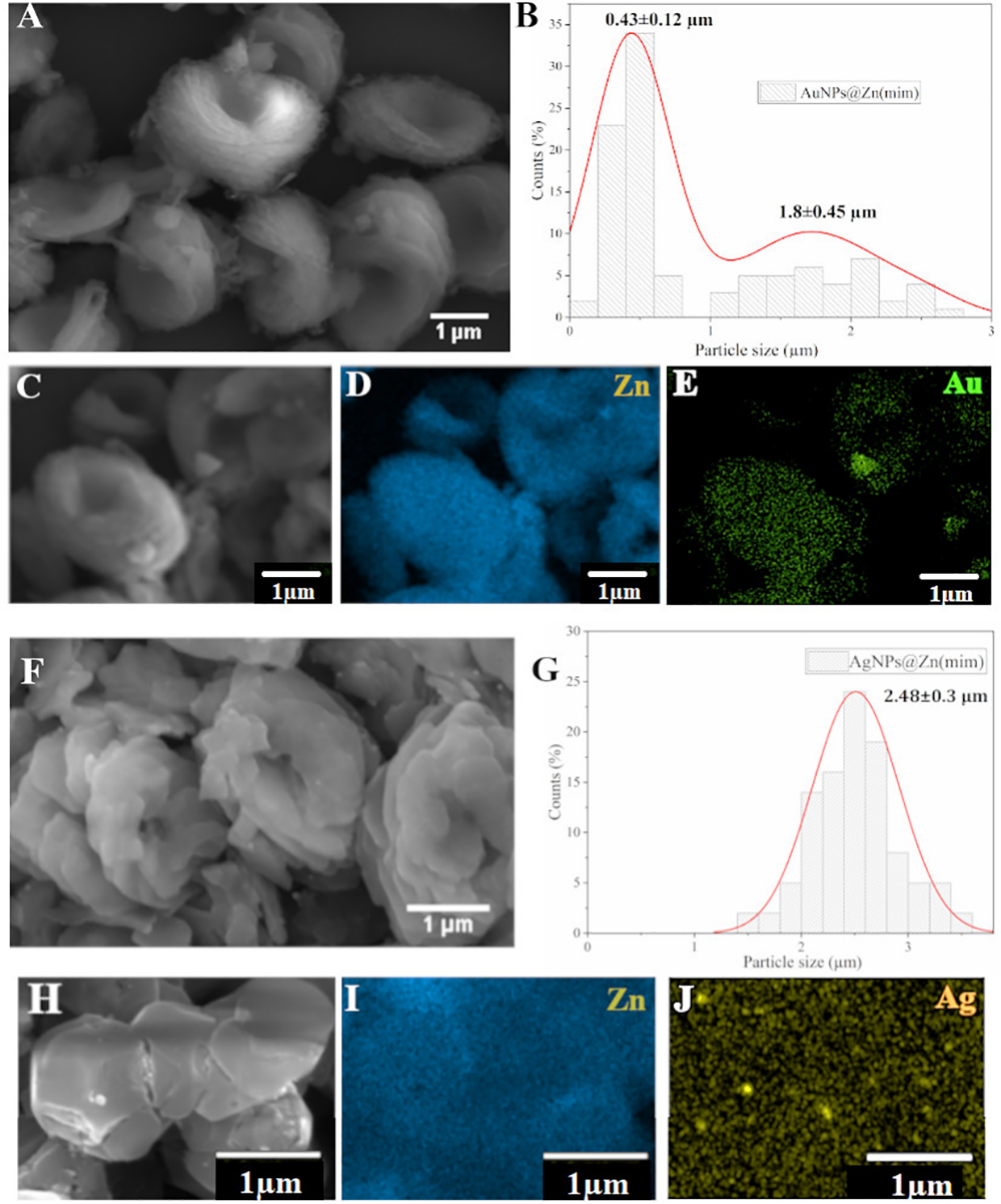
SEM images, histograms, and SEM-EDS mappings of samples AuNPs@Zn(mim) (A–E) and AgNPs@Zn(mim) (F–J).

The morphology of the prepared nanomaterials was evaluated by SEM analysis (Figure 4).

The micrographs of the Zn support (mim), Figure S5, exhibit a predominantly polydisperse truncated rhombic dodecahedron morphology^42^ with average particle sizes of 0.94 ± 0.21 μm. For the support of the AuNPs@Zn(mim) sample, two different morphologies were identified. The first morphology is composed of smaller, more rounded particles (similar to ZIF-8, indicated with red arrows), and the second one is composed of multilayer plates with average particle sizes of 0.43 ± 0.12 and 1.8 ± 0.45 μm, respectively (Figure 4A, B, F, and G). For the AgNPs@Zn(mim) sample support, multilayer plate-like morphology aggregates were found with an average size of 2.48 ± 0.3 μm (Figure 4F,G). The analyzed materials presented morphological aspects in agreement with those described in the literature, where the truncated rhombic dodecahedron phase is the main for ZIF-8^42^ and the bigger aggregates of the multilayer plate-like phase are characteristic of dia(Zn).^49,50^

The elemental mapping with energy-dispersive microscopy (EDS) for AuNPs@Zn(mim) and AgNPs@Zn(mim) samples, Figure 4D, E, I, and J, indicates that Zn, Au, and Ag are uniformly distributed, and no clusters are observed.

#### 3.1.7 TEM

**Figure 5:**
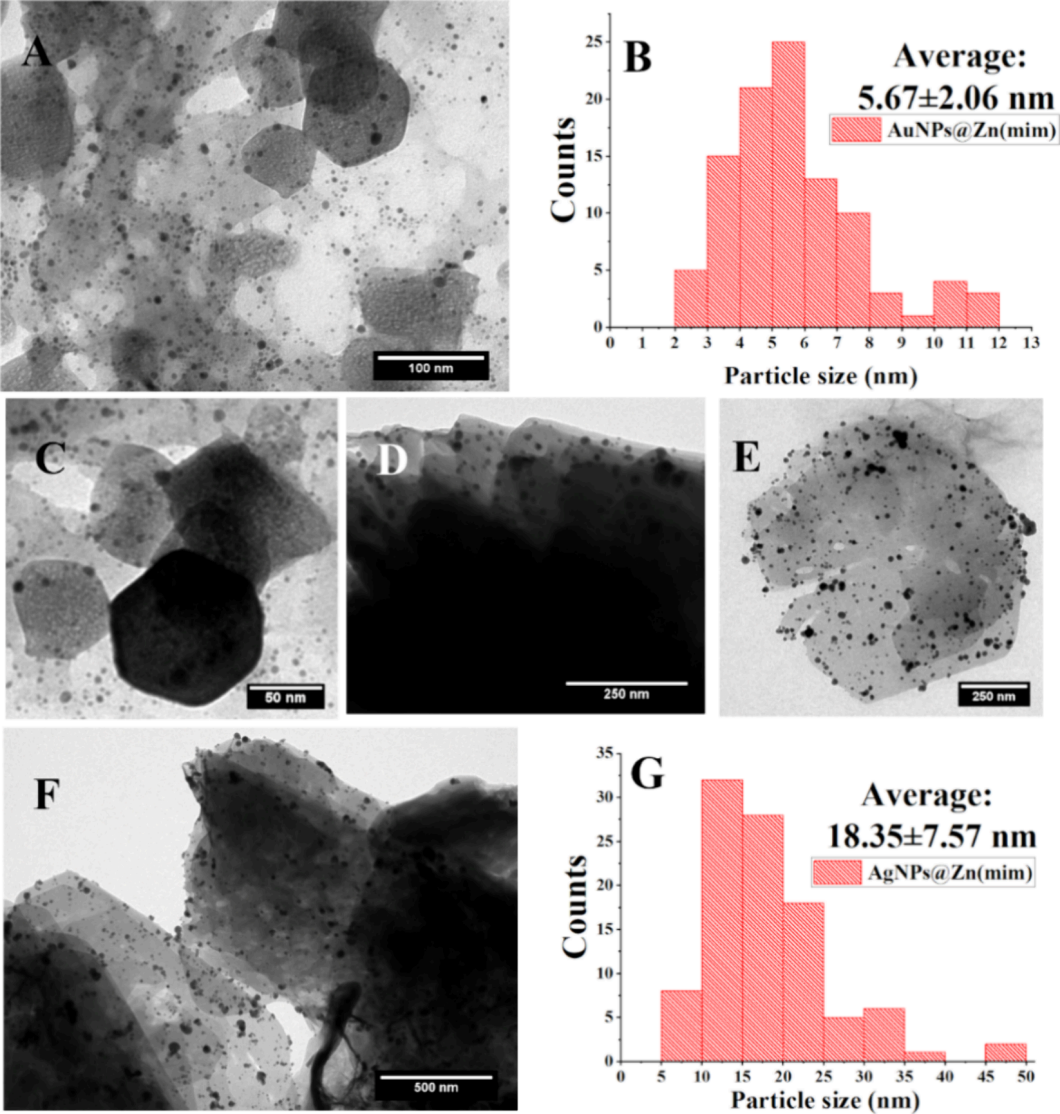
TEM images and size distribution of particle samples of AuNPs@Zn(mim) (A–C) and AgNPs@Zn(mim) (D–G).

To assess the size and shape of MNPs on the Zn(mim) support, TEM images were collected (Figure 5).

From the TEM images in Figure 5, it is possible to observe that the MNPs (AuNPs or AgNPs) are well dispersed on the support Zn (min). The size distribution of these nanoparticles (Figure 5B,G), counting 100 individual nanoparticles from a set of three TEM images, showed average sizes of 5.67 ± 2.06 and 18.35 ± 7.57 nm for AuNPs@Zn(min) and AgNPs@Zn(min), respectively.

### 3.2 Catalytic Activity: Hydrogen Production

Initially, a dehydrogenation experiment was conducted using the AgNPs@Zn(mim) catalyst at near-ambient temperature, 30 °C. However, it was found that only 18.86% gas was generated during 34.7 min, resulting in a production of 9 mL of H_2_. Considering this result, we adjusted the temperature to 40 °C to continue the studies.

**Figure 6:**
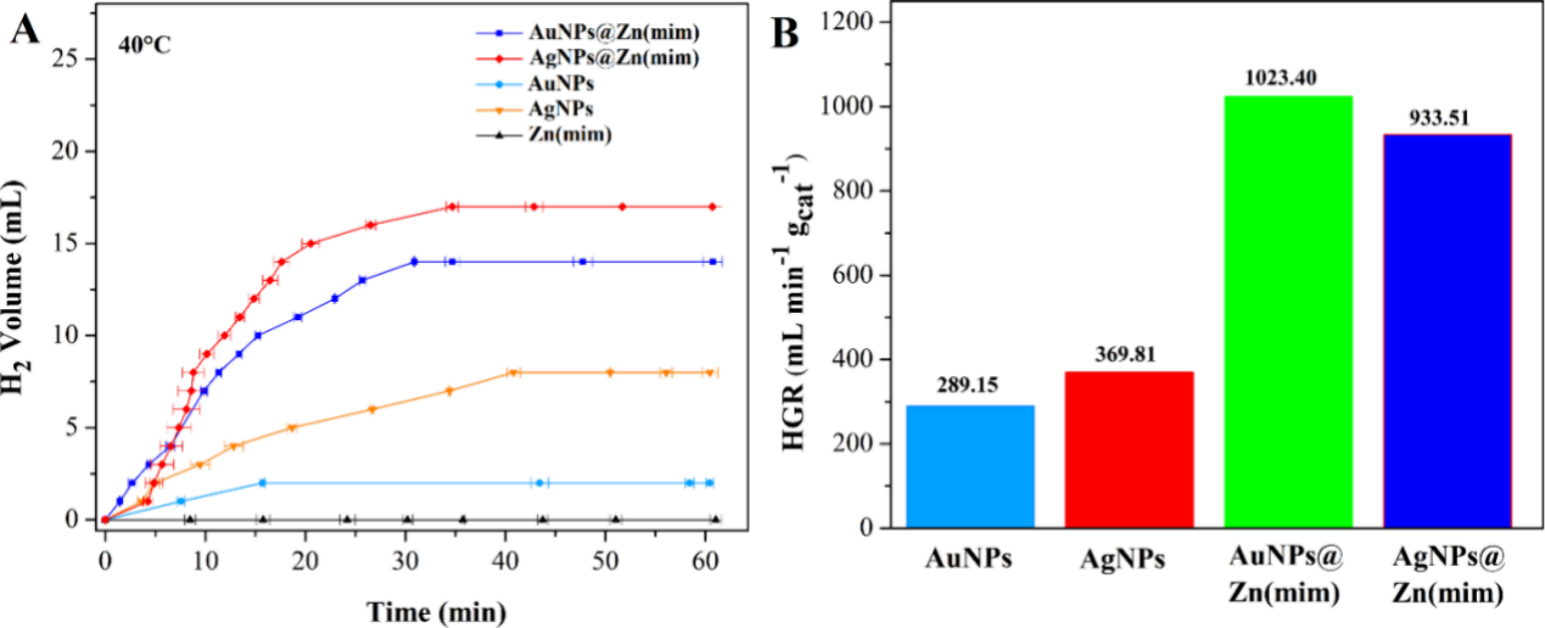
Experiments of hydrogen generation from hydrolysis of NH_3_BH_3_ using nanoparticles (AuNPs and AgNPs) and metal–organic support [Zn(mim)] compared to the catalytic activity of the composites (AuNPs@Zn(mim) and AgNPs@Zn(mim)) at a temperature of 40 ± 0.2 °C. Volume of hydrogen produced (A) and HGR (B).

The formation of hydrogen through the hydrolysis of NH_3_BH_3_ was investigated with the performance of control experiments. In the absence of catalysts, no gas formation was observed at 40 and 50 °C during 60 min of the dehydrogenation reaction (Figure S6). At temperatures of 60 and 70 °C, 1 and 3 mL of hydrogen were produced, respectively, during 60 min, which represents about 2.1 and 6.3% of the yield, respectively. The addition of the support [Zn(mim)] to the system did not significantly increase the yield of H_2_ production (Figure S7). To verify if the nanoparticles (AuNPs and AgNPs) present some catalytic activity, NH_3_BH_3_ was hydrolyzed at 40 °C (Figure 6).

The total volume of hydrogen produced using AgNPs was four times higher than for AuNPs, namely, 8 mL (369.81 mL min^–1^ g_cat_^–1^/ 16.76% yield of H_2_) vs 2 mL (289.15 mL min^–1^ g_cat_^–1^/ 4.19% yield of H_2_), respectively. However, the reaction time was more than two times longer than in the case of AuNPs (40.8 vs 15.7 min for AgNPs and AuNPs, respectively).

AuNPs@Zn(mim) and AgNPs@Zn(mim) proved to be highly active catalysts in the production of H_2_ from NH_3_BH_3_ hydrolysis (Figure 6). Compared with colloidal NPs, the HGR values determined for the reactions catalyzed by AgNPs@Zn(mim) and AuNPs@Zn(mim) were more than 2.5 and 3 times higher, respectively. A slightly better yield but a longer reaction time was achieved in the case of AgNPs@Zn(mim) (35.62% yield of H_2_ in 34.7 min) compared to AuNPs@Zn(mim), which produced 14 mL in 30.88 min (29.24% yield of H_2_). This result indicates that the synergistic effect of the metal–organic support with the nanoparticles resulted in a catalytic improvement. The values of the HGR, TOF, and yield (%) are presented in Table S1.

#### 3.2.1 Effect of Reaction Temperature

**Figure 7:**
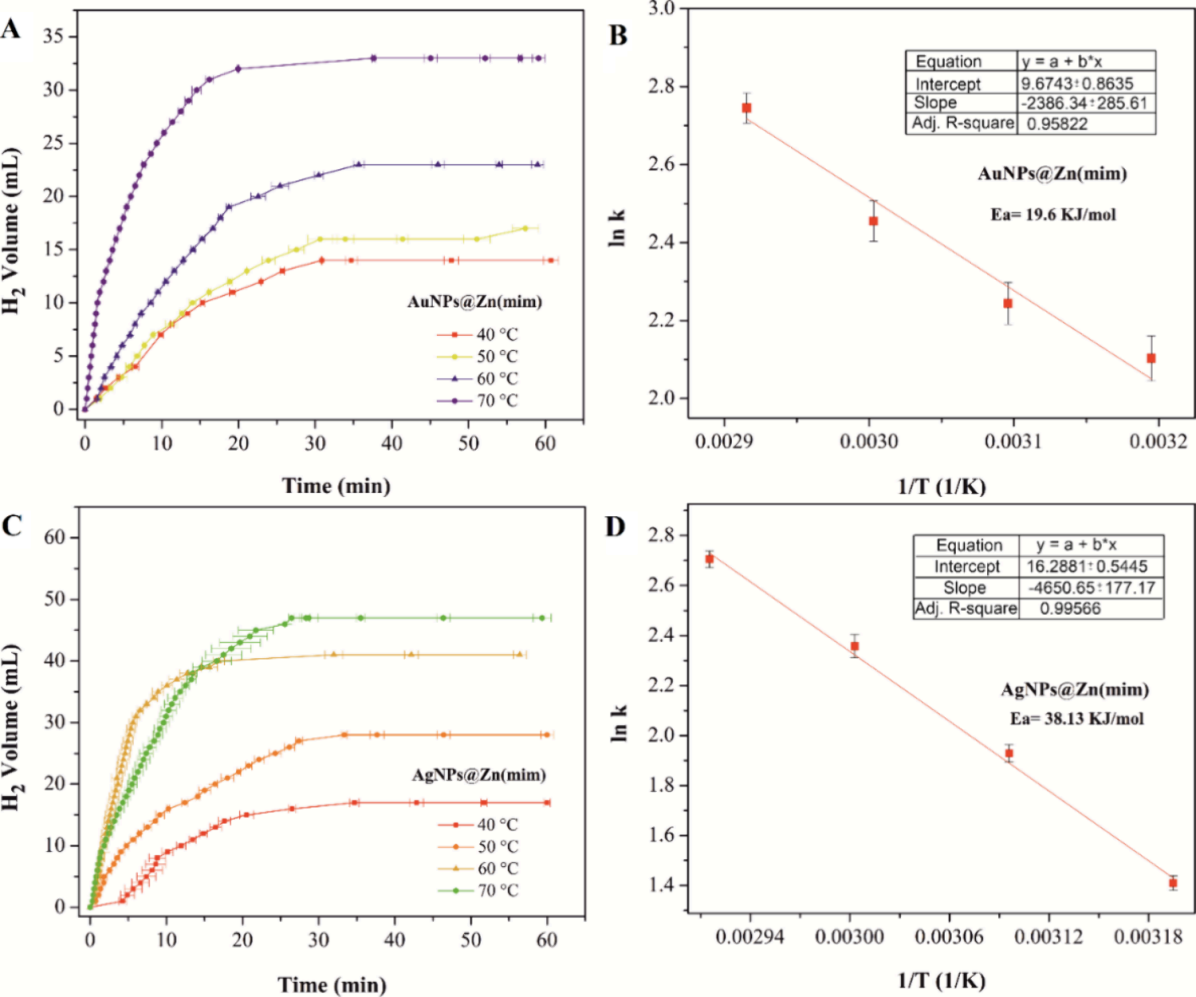
Temperature influence on hydrogen generation from NH_3_BH_3_ catalyzed by AuNPs@Zn(mim) (A) and AgNPs@Zn(mim) (C). Arrhenius plots determined for the hydrolysis catalyzed by AuNPs@Zn(mim) (B) and AgNPs@Zn(mim) (D).

Figure 7 depicts the effect of the reaction temperature on hydrogen generation. The reaction rate and yield increased as the reaction temperature increased (Table S2).

The analysis of the change in hydrogen volume over time indicates that the most suitable model for interpreting the results is the zero-order model (Table S3 and Figure S8), where the reaction rate is not affected by variations in the concentration of reactants. The value of the kinetic constant determined at 40 °C for the AgNPs@Zn(mim) catalyst was higher than that for the AuNPs@Zn(mim)-catalyzed system.

With increased temperature, an increase in HGR and TOF values was observed for the system catalyzed by both catalysts (Table S2). In all cases, except for the temperature of 40 °C, the obtained results were better for AgNPs@Zn(mim) than for the Au-bearing counterpart (by a factor of up to 1.7 times). On the other hand, the same comparison cannot be made with TOF, which presented higher values for the AuNPs@Zn(mim) catalyst. The best values of TOF, HGR, and yield were obtained with the catalyst AgNPs@Zn(mim), which has 1.06 wt % silver, reaching yields of 98.5% of the hydrogen produced in 26.45 min at 70 °C.

The activation energy was calculated from the slope of the Arrhenius graph (Figure 7B,D). The value determined for the AuNPs@Zn(mim)-catalyzed system was lower than in the case when AgNPs@Zn(mim) was used as the catalyst (19.60 kJ mol^–1^ vs 38.13 kJ mol^–1^, respectively). These values are comparable to those of catalysts containing at least one supported metal used in the same reaction (Table 3).

**Table 3:** Comparison of the Activation Energy and Catalytic Activity of Various Supported Catalysts in the Hydrolysis of NH_3_BH_3_^a^

	metal (wt %)	TOF (molH_2_ min^–1^ mol_Metal_)^−1^	*E*_a_ (kJ mol ^–1^)	HGR mL (min g_cat_) ^–1^	ref.
AgPd @UIO-66-NH_2_	1.00 (Ag) and 4.00 (Pd)	90.00	51.77	NI	(28)
Ag@Ni/graphene	NI	77.00	49.56	NI	(53)
Au/NiP	2.21 (Au)	1360.3	45.28	68.3	(54)
AgCo/gC_3_	4.20 (Ag) e 20.10 (Co)	249.02	40.91	NI	(55)
**AgNPs@Zn(mim)**	**1.06**	**14.71**	**38.13**	**3352.71**	**this work**
AuNi@ZIF-8	6.9 (Au)	2.40	37.4	NI	(30)
Au@Cu_2_O	1.42 (Au) and 55.83 (Cu)	1.41	28.4	NI	(56)
**AuNPs@Zn(mim)**	**0.89**	**15.84**	**19.60**	**1979.53**	**this work**
Au/γ-Al_2_O_3_	NI	no activity	NI	no activity	(57)
AuCo@MIL-101	6 0.00 (Au) and 94.00 (Co)	23.5	NI	NI	(29)
Ag@Zn-MOF	NI	NI	NI	NI	(32)
AuCo@ZIF-8	6.0	40.0	NI	NI	(30)

a NI, not informed.

The comparison of two tested catalysts, AgNPs@Zn(mim) and AuNPs@Zn(mim), shows that the former is more active in NH_3_BH_3_ hydrolysis. Even though the activation energy was lower for the reaction with AuNPs@Zn(mim), the HGR and volumetric yield values were higher for the AgNPs@Zn(mim)-catalyzed system (Table S2). Additionally, the synthesis of the Ag-bearing material is more cost-effective. Thus, this catalyst was used for further investigation.

Yüksel et al. highlight the direct correlation between the amount of released ammonia and the concentration of the NH_3_BH_3_ solution, noting that concentrations greater than 15% by weight have a substantial relationship with the amount of ammonia produced.^18^ Thus, the purity of H_2_ was related to the absence of ammonia during the dehydrogenation of NH_3_BH_3_. After the end of hydrogen evolution (approximately 60 min), an aqueous solution of CuSO_4_ was analyzed for precipitates. No precipitates were observed, indicating the absence of ammonia. In contrast, a reference test using NH_4_OH in CuSO_4_ resulted in the formation of a light-blue precipitate, identified as copper(II) hydroxide. Excess NH_3_ in the same reaction formed an intense-blue soluble salt complex, associated with tetraamminecopper(II) sulfate (Figure S9),^58^ as evidenced by the chemical equations provided in the supporting material. These findings are in agreement with the UV–vis spectra, where the precipitate band formed in the reference experiment (602 nm) is evident, while in the experiment solution with the catalyst, only the CuSO_4_ band was observed (above 800 nm).^58^

#### 3.2.2 Effect of NaOH Concentration on the Reaction

**Figure 8:**
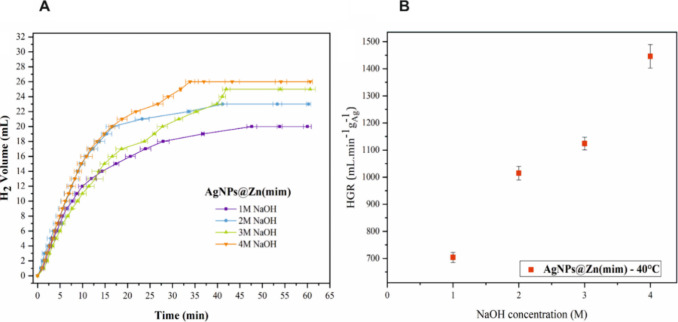
Effect of NaOH concentration on (A) produced hydrogen volume and (B) HGRs determined in NH_3_BH_3_ hydrolysis catalyzed by AgNPs@Zn(mim).

In the next step, the effect of NaOH concentration on hydrogen production via NH_3_BH_3_ hydrolysis was tested (Figure 8).

Initially, it can be observed that the HGR (Table S4) decreased considerably with the addition of 1 M NaOH. The hydrogen production rate increased with increasing NaOH concentrations from 1 to 4 M. Compared to the reaction without NaOH, the addition of the base accelerated the hydrolysis. The HGR value increased from 933.51 mL min^–1^ g_Ag_^–1^ without NaOH to 1445.82 mL^–1^ min g_Ag_^–1^ in the presence of 4 M NaOH. Yao et al.^59^ noted that no H_2_ release is observed for NH_3_BH_3_ in 1 M NaOH solution without the catalyst, indicating that NaOH acts as a catalyst promoter for the hydrolytic dehydrogenation of NH_3_BH_3_. According to the literature, OH^–^ ions act as cocatalysts to activate water molecules. The improvement of the catalytic activity is due to the OH^–^ ion coordination on the metal nanoparticle surface, enriching these NPs in electrons, which favors the oxidative action of water (cleavage of the O–H bond of H_2_O) and the NH_3_BH_3_ molecule activation.^59,60^ However, excess coordination can occupy the active sites of the metal surface, partially inhibiting the oxidative action of the water OH bond, leading to a decrease in the hydrolysis rate.^60^

In general, the addition of NaOH in the catalytic dehydrogenation of NH_3_BH_3_ has a significant effect on the reaction rate.^54,61^ The equilibrium of the reaction can be shifted toward dehydrogenation, increasing the release of hydrogen. The literature highlights that there is an optimal concentration of NaOH that maximizes catalytic activity,^62−64^ which varies from one catalyst to another.^54^

Although the introduction of the base has demonstrated notable impacts on the HGR, it is essential to recognize that in excess the base can saturate the catalyst surface. This saturation, in turn, tends to unfavorably affect the cleavage of water and the efficient activation of the NH_3_BH_3_ molecule, resulting in a decrease in the promotion caused by NaOH in the rate of hydrolysis, especially as concentrations increase. This phenomenon becomes evident in the results for concentrations of 3 and 4 M, where the observation of a significant increase in the hydrogen rate becomes less pronounced.

Moreover, the addition of the base can also decrease the concentration of NH_4_^+^ in an aqueous solution (due to the reaction: NH_4_^+^ + OH^–^ → NH_4_OH) and accelerate the equilibrium shift to the product side (NH_3_BH_3_ + 2H_2_O → NH_4_^+^+ BO_2_^–^+3H_2_). On the other hand, increasing the concentration of NH_4_^+^ can significantly decrease the catalytic activity.^59^

**Figure 9:**
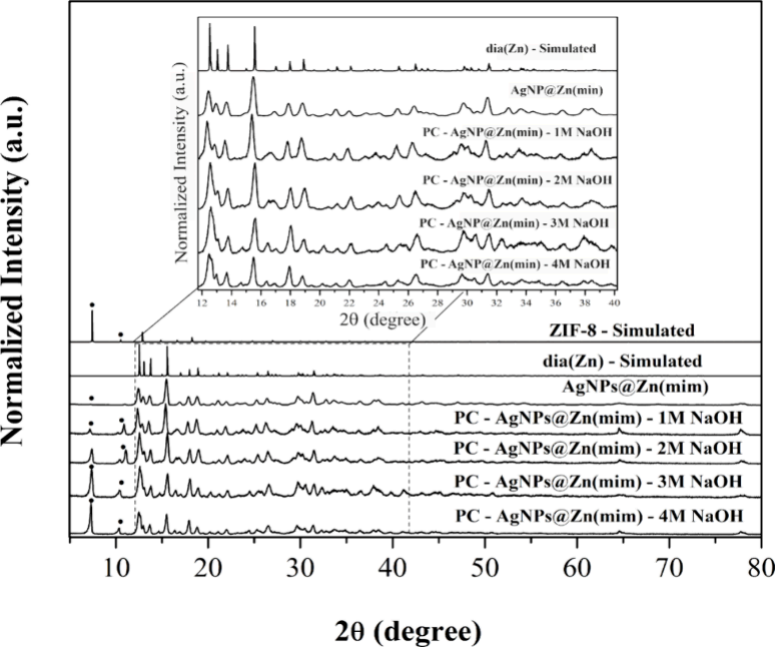
Diffractograms of the postcatalysis AgNPs@Zn(mim) isolated from NH_3_BH_3_ hydrolysis carried out with different NaOH concentrations (1–4 M).

After catalytic tests with NaOH, the AgNPs@Zn(mim) catalyst was characterized by the XRD technique, as shown in Figure 9.

After the catalytic reaction was carried out under alkaline conditions, some structural changes in the diffractograms of AgNPs@Zn(mim) were observed. Before basic catalysis, the AgNPs@Zn(mim) catalyst showed a predominantly dia(Zn) phase (Figure 2). In the presence of NaOH, it was observed that the amount of the dia(Zn) phase decreased, and the ZIF-8 phase was favored.

Based on the XRD analysis, the crystallite size was calculated in relation to the dia(Zn) phase. Compared with the catalyst isolated from the reaction without NaOH, smaller crystallite sizes were obtained for lower NaOH concentrations (Table S5).

SEM micrographs of postbasic catalysis samples (Figure S10A–H) clearly show that they maintained their original morphology of clusters of rounded plates in multilayers of the dia(Zn) crystalline phase^49,50^ and other smaller particles.

In the FTIR spectrum of the AgNPs@Zn(mim) catalyst (Figure S11) used in the reaction with 3 and 4 M NaOH, a low-intensity broad band approximately at 3400 cm^–1^ is observed, which is related to bonded hydroxyl groups.^39^

#### 3.2.3 Reuse: Lifetime

Catalyst durability (lifetime) is a necessary and crucial parameter for practical application, which can effectively reflect the catalyst stability to retain its initial activity in continuous operation under reaction conditions. Furthermore, the stability of metal centers can also influence catalyst durability, where agglomeration of metallic NPs, structure collapse, and loss of active components often lead to a rapid decrease in catalytic activity.^16^

The lifetime test was carried out for the two catalysts AuNPs@Zn(mim) and AgNPs@Zn(mim). To assess the reusability of the materials, the catalysts after NH_3_BH_3_ hydrolysis were used again without regeneration in the following reaction cycle.

**Figure 10:**
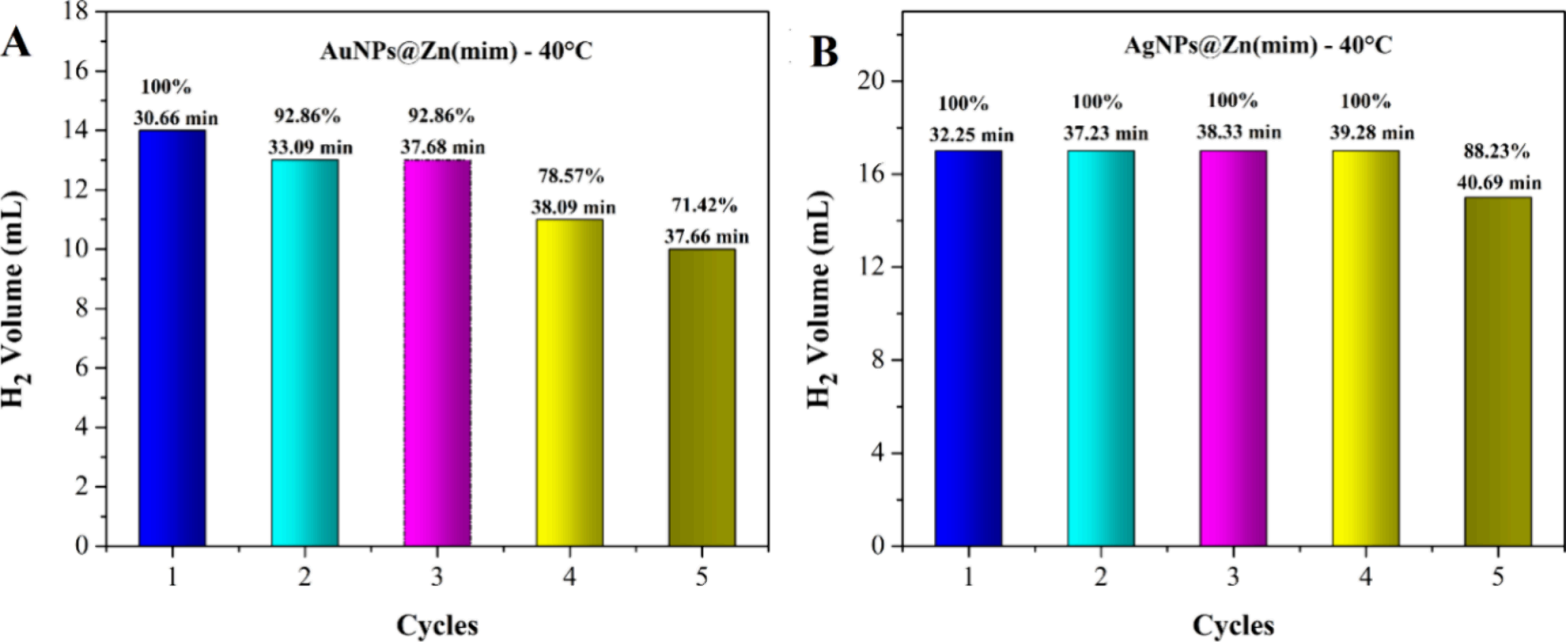
H_2_ volume in five consecutive reaction cycles using (A) AuNPs@Zn(mim) and (B) AgNPs@Zn(mim).

The catalysts AuNPs@Zn(mim) and AgNPs@Zn(mim) were still active after five reaction cycles, retaining about 71.42 and 88.23%, respectively, of the initial catalytic activity of hydrogen generation in the hydrolysis of NH_3_BH_3_ (Figure 10). High recyclability may suggest low leaching and/or less catalyst deactivation.

The AuNPs@Zn(mim) catalyst gradually decreases the HGR at each cycle (Table S6). On the other hand, the AgNPs@Zn(mim) catalyst shows a considerable decrease in the H_2_ generation rate from the first to the second cycle; then, from the second to the fourth cycle, there is no significant change in the rate of H_2_ generation; and finally, from the fourth to the fifth cycle, there is a second significant decrease in the H_2_ generation rate. Therefore, even though the AuNPs@Zn(mim) catalyst presents a higher H_2_ generation rate than the AgNPs@Zn(mim) catalyst (Table S6), the catalyst with AgNPs, under the same reaction conditions, shows greater stability over the cycles and higher values of H_2_ production yields.

The decrease in the H_2_ generation efficiency is probably related to the reduction in the accessibility of the active sites of the nanoparticles. Furthermore, the decrease in catalytic activity in successive runs can also be attributed to the aggregation of nanoparticles on the surface of the support.^65,66^ In general, the crystallite sizes of the studied catalysts decreased after five cycles of catalysis (Table S5).

The reused AuNPs@Zn(mim) and AgNPs@Zn(mim) catalysts were characterized after the fifth run by the XRD technique (Figure S12).

After five cycles of the reaction, the diffractograms showed no significant change in the phase percentage of the two catalysts, AuNPs@Zn(mim) and AgNPs@Zn(mim). However, the increase in baseline thickness is perceptible, which may be related to the increase in the amorphous phase of the material and to impurities (catalysis byproducts). The most prominent change is observed in the increase in the relative intensity of the crystallographic planes related to silver and gold nanoparticles, shown by asterisks (*) in Figure S12, probably due to agglomeration, which causes the increase in the size of these MNPs.

In SEM micrographs of the postcatalysis materials, the morphology of clusters of rounded plates in multilayers of the dia(Zn) crystalline phase can still be observed (Figure S10I,J).^49,50^ Nevertheless, the prevalence of smaller particles can be attributed to the delamination of the multilayer material.

## 4 Conclusions

The AuNPs@Zn(mim) and AgNPs@Zn(mim) catalysts were prepared in a simple, environmentally friendly way, without using organic solvents and at room temperature. Incorporating MNPs into the Zn(mim) support did not considerably alter the thermal stability of the catalysts. However, the change from Au to Ag resulted in a change in the amount of the dia(Zn) crystalline phase.

Both catalysts showed excellent catalytic activities in the hydrolysis of NH_3_BH_3_, with a maximum TOF of 15.84 mol_H2_ (mol_Au_ min)^−1^ for AuNPs@Zn(mim) and 14.71 mol_H2_ (mol_Ag_ min)^−1^ for AgNPs@Zn(mim). The hydrogen yield was 69.16% for AuNPs@Zn(mim) and 98.50% for AgNPs@Zn(mim) at 70 ± 0.2 °C, with HGRs of 1979.53 mL (min gAu)^−1^ and 3352.71 mL (min gAg)^−1^, respectively. The introduction of NaOH increased the hydrolysis rate catalyzed by AgNPs@Zn(mim) at 40 ± 0.2 °C. Furthermore, the AuNPs@Zn(mim) and AgNPs@Zn(mim) catalysts demonstrated good reusability, maintaining 71.42% and 88.23% of the initial activity after five cycles.

The results highlight the synergistic effect of the metal–organic support and metal nanoparticles on the catalytic efficiency. AgNPs@Zn(mim) was superior to AuNPs@Zn(mim) in terms of activity and cost, offering a promising alternative for practical applications. Future studies should explore the optimization of these catalysts to further improve their performance and their evaluation in other hydrogen-release reactions.

## Supporting Information Available

Equations for TOF, HGR, yield, and activation energy calculations, crystallite size calculations, and synthesis of MNPs by the chemical reduction method in addition to Figures S1–S13 and Tables S1–S6 (PDF)
