# Supplementary material for: Fabrication of Gold and Silver Nanoparticles Supported on Zinc Imidazolate Metal–Organic Frameworks as Active Catalysts for Hydrogen Release from Ammonia Borane
Source: ACS Omega. 2024 Sep 18;9(39):41084–96. doi: 10.1021/acsomega.4c07068 (PMC11447819; doi:10.1021/acsomega.4c07068)
Supplement: Supplementary file 1 — ao4c07068_si_001.pdf [file ao4c07068_si_001.pdf]

Supporting Information

Fabrication of gold and silver nanoparticles supported on zinc imidazolate metal-organic frameworks as active catalysts for hydrogen release from ammonia borane

Elibe S. Souza[a#], Maria Alaide de Oliveira[b], Jildimara de Jesus Santana[a], Natalia Łukasik[b$], Ohanna Maria Menezes Madeiro da Costa[c], Luciano Costa Almeida[d], Bráulio Silva Barros[e,*], and Joanna Kulesza[f,*]

[a] *Programa de Pós-graduação em Ciência de Materiais, Centro de Ciências Exatas e da Natureza-CCEN, Universidade Federal de Pernambuco, Recife - PE, Avenida Jornalista Aníbal Fernandes, s/nº, Cidade Universitária, 50740-560, Brasil.*

[b] *Programa de Pós-graduação em Química, Centro de Ciências Exatas e da Natureza-CCEN, Universidade Federal de Pernambuco, Recife – PE, Avenida Jornalista Aníbal Fernandes, s/nº, Cidade Universitária, 50740-560, Brasil.*

[c] *Brazilian Synchrotron Light Laboratory (LNLS), Brazilian Center for Research in Energy and Materials (CNPEM), Rua Giuseppe Máximo Scolfaro, Cidade Universitária, 13083-100 Campinas, SP, Brazil.*

[d] *Departamento de Engenharia Química, Centro de Tecnologia e Geociências - CTG, Universidade Federal de Pernambuco, Recife – PE, Rua Artur de Sá, Cidade Universitária, 50740-521, Brasil.*

[e] *Departamento de Engenharia Mecânica, Centro de Tecnologia e Geociências - CTG, Universidade Federal de Pernambuco, Recife – PE, Av. Prof. Morais Rego, 1235- Cidade Universitária, 50670-901, Brasil.*

[f] *Departamento de Química Fundamental, Centro de Ciências Exatas e da Natureza-CCEN, Universidade Federal de Pernambuco, Recife – PE, Av. Prof. Morais Rego, 1235- Cidade Universitária, 50670-901, Brasil.*

*Email: joanna.kulesza@ufpe.br

*Email: braulio.barros@ufpe.br

Present addresses:
[#]E. S. S: Programa de Pós-graduação em Química, Centro de Ciências Exatas e da Natureza-CCEN, Universidade Federal de Pernambuco, Recife – PE, Avenida Jornalista Aníbal Fernandes, s/nº, Cidade Universitária, 50740-560, Brasil
[$]Natalia Łukasik: Department of Process Engineering and Chemical Technology, Faculty of Chemistry, Gdańsk University of Technology, 11/12 Narutowicza Street, 80-233 Gdańsk, Poland.

Hydrogen generation tests

The parameters describing the catalyst efficiency in the hydrolysis reaction were evaluated based on the equation:

- The turnover frequency (TOF):

$$TOF = \frac{nH_2}{t.M_{cat}}$$

n_{H2} denotes the mole number of generated hydrogen (mole), *t* denotes the corresponding hydrolysis time (min), and $M_{Cat.}$ denotes the number of active sites in the catalyst used in the catalytic tests (moles), determined from IPC-OES analysis.

- the hydrogen generation rate (HGR): $HGR = \frac{V_{H_2}}{t \times m_{cat}}$ where: V_{H_2} is a volume of hydrogen

generated during NH_3NH_3 hydrolysis (mL), *t* is the total time in the maximum volume of hydrogen generation (min), m_{cat} is the mass of metal of the catalyst (g) determined by ICP-OES.

- The yield value can be calculated by the following equation:

$$yield = \frac{V_{generated\, H_2}}{V_{theoretical\, H_2}} \times 100\%$$

where $V_{generated\, H_2}$ is the total volume of generated H_2 (L), and $V_{theoretical\, H_2}$ is the theoretical volume of H_2 (L) that can be produced via NH_3BH_3 hydrolysis under reaction conditions.

- The activation energy (Ea) of the reaction was evaluated following the reaction procedure at different temperatures, keeping the constant concentrations of NH_3BH_3 and catalyst. Ea can be calculated from the following Arrhenius equation:

$$lnk_0 = \frac{-E_a}{RT} + lnA$$

where k_0 denotes the rate constant, R is the ideal gas constant (8.314 J mol^{-1} K^{-1}), T represents the reaction temperature (K), and A is the pre-exponential factor.

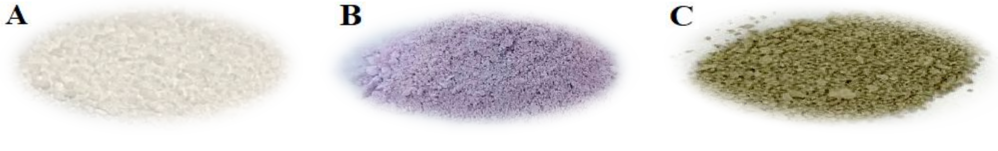

Figure S1 Powders of the obtained samples. A) Zn(mim); B) AuNPs@Zn(mim) e C) AgNPs@Zn(mim).

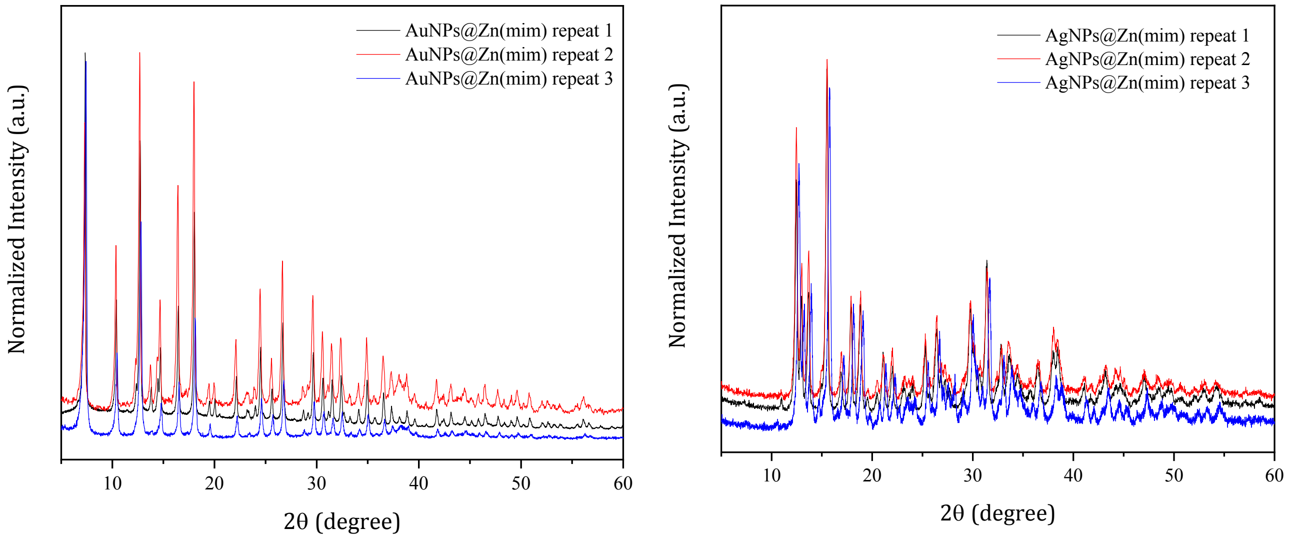

Figure S2 PXRD patterns of MNPs@Zn(mim) samples demonstrating the reproducibility of the synthesis of materials in different repetitions.

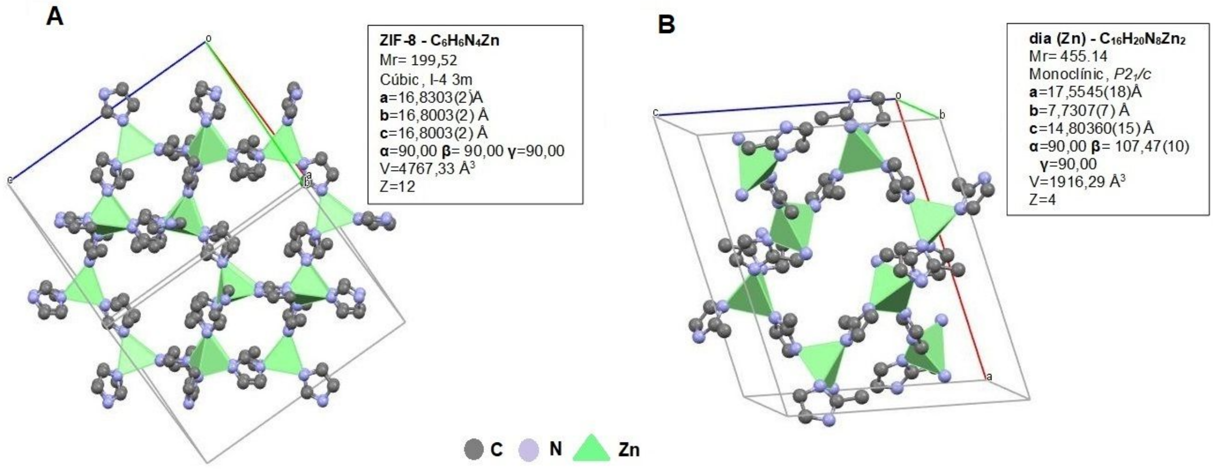

Figure S3 Simulated crystalline structures: A) ZIF-8 [1] and B) dia(Zn) [2].

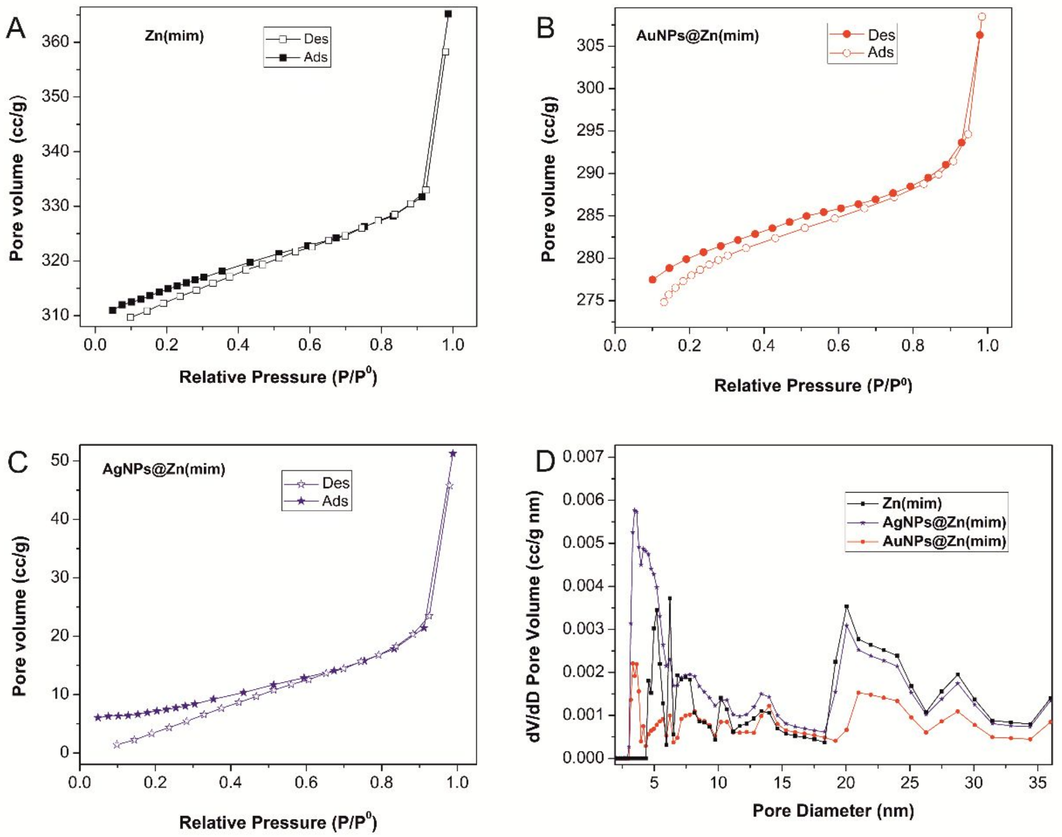

Figure S4 N_2 absorption-desorption isotherm curves (A-C) and pore size distribution curve (D) of the prepared samples.

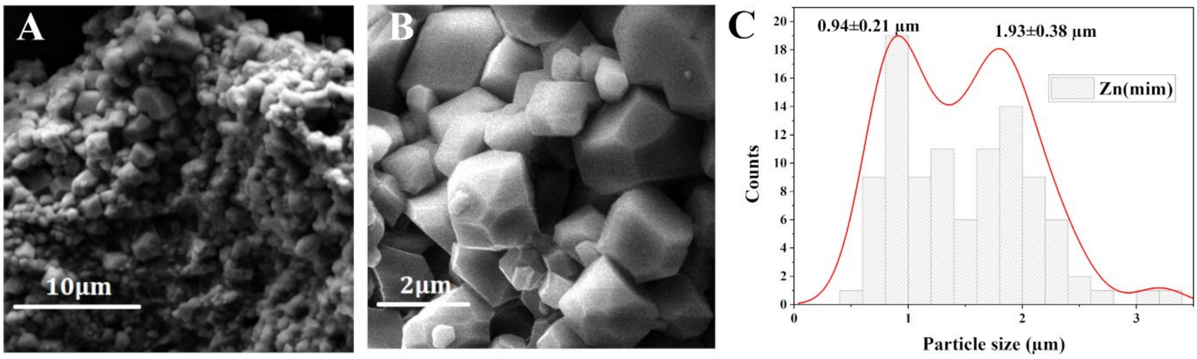

Figure S5 - SEM images and histograms of sample Zn(mim) (A-C).

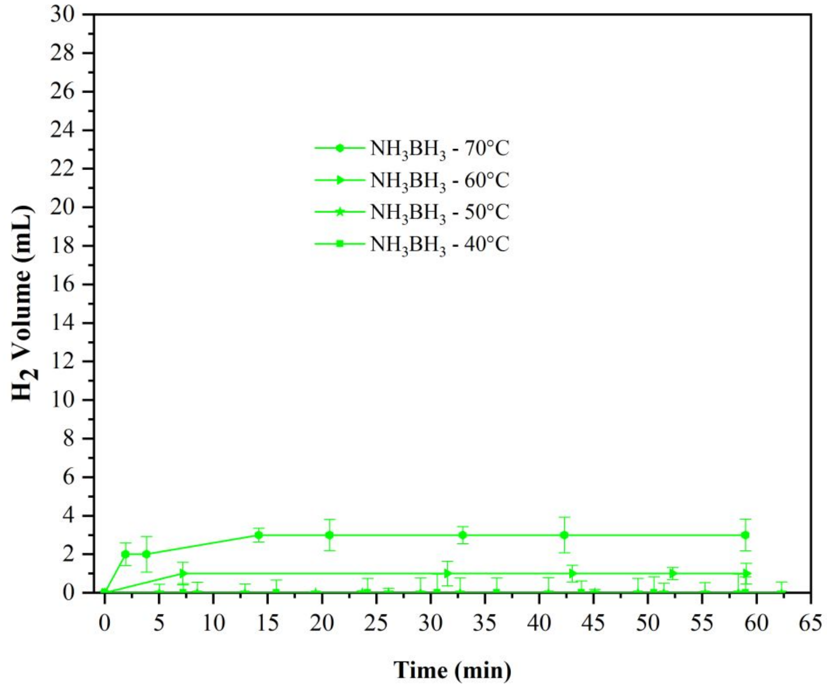

Figure S6 Hydrogen generation via NH_3BH_3 hydrolysis without a catalyst at 40, 50, 60, and 70 ±0.2 °C.

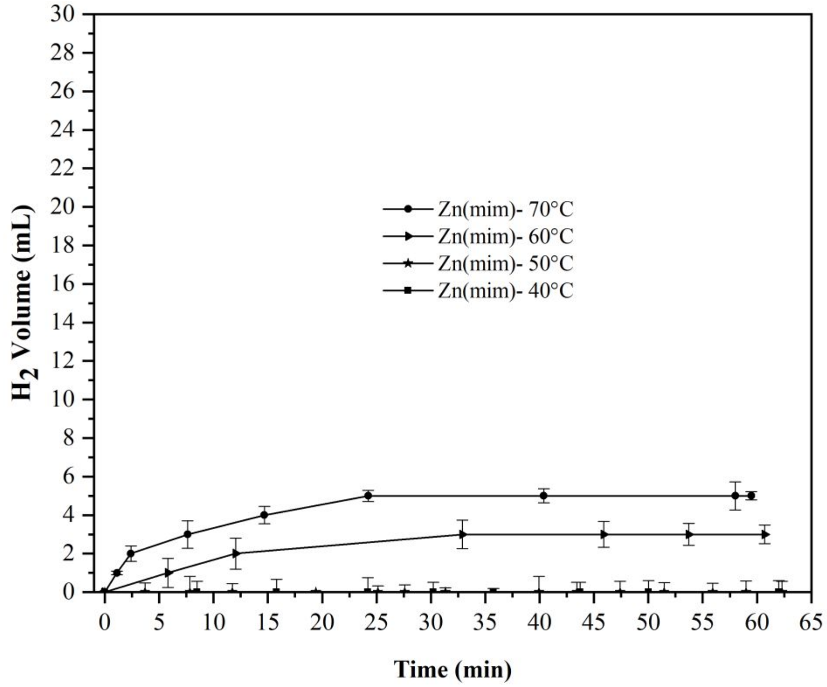

Figure S7 Hydrogen generation via NH_3BH_3 hydrolysis in the presence of the Zn(mim) support at 40, 50, 60, and 70 ±0.2 °C.

Table S1 Turnover frequency (TOF), hydrogen generation rate (HGR), and hydrogen yield for experiments of catalytic activity with nanoparticles (AuNPs and AgNPs), metalorganic support [Zn(mim)], AuNPs@Zn(mim) and AgNPs@Zn(mim)catalysts in NH_3BH_3 hydrolysis at a

temperature of 40±0.2 °C.

**Sample**	**TOF** mol H_2.mol$_{cat}^{-1}$. min^{-1}	**HGR** mL.min^{-1}. g$_{cat}^{-1}$	**Yield** (%)	**Time** (min)	**Volume** (mL)
AuNPs (1wt%)	2.21	289.15	4.19	15.7	2
AgNPs (1wt%)	1.60	369.81	16.76	40.8	8
AuNPs@Zn(mim)	8.19	1023.40	29.34	30.88	14
AgNPs@Zn(mim)	4.10	933.51	35.62	34.70	17
Zn(mim)	0	0	0	61	0

Table S2 Turnover frequency (TOF), hydrogen generation rate (HGR), and hydrogen yield for AuNPs@Zn(mim) and AgNPs@Zn(mim) catalysts for NH_3BH_3 hydrolysis with temperature variation.

**T** (°C)	**TOF** mol H_2.mol$_{cat}^{-1}$. min^{-1}	**HGR** mL.min^{-1}. g$_{cat}^{-1}$	**Yield** (%)	**Time** (min)	**Volume** (mL)
**AuNPs@Zn(mim)**					
40	8.19	1023.40	29.34	30.88	14
50	9.43	1178.38	33.53	30.65	16
60	11.65	1455.53	48.19	35,67	23
70	15.84	1979.53	69.15	37.63	33
**AgNPs@ Zn(mim)**					
40	4.10	933.51	35.62	34.70	17
50	6.95	1583.16	58.67	33.37	28
60	10.61	2417.45	85.91	32.00	41
70	14.71	3352.71	98.50	26.45	47

Table S3 The kinetic parameters of hydrogen generation via catalyzed NH_3BH_3 hydrolysis at 40±0.2°C.

Kinetic order	Parameter	AuNPs@Zn(mim)	AgNPs@Zn(mim)
zero	k	0.7025	1.3343
	R^2	0.9898	0.9964
first	k	0.1918	0.3453
	R^2	0.9189	0.8965
second	k	-0.08574	-0.1208
	R^2	0.6216	0.6517

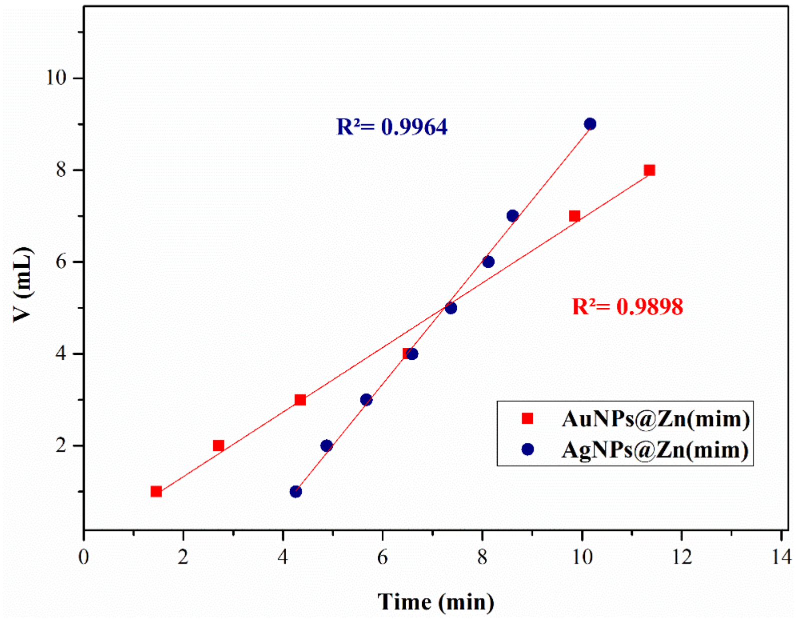

Figure S8 Time vs evolution of H_2 in mL for the catalysts AuNPs@Zn(mim) and AgNPs@Zn(mim), demonstrating that the most suitable hydrogen generation reaction is zero-order.

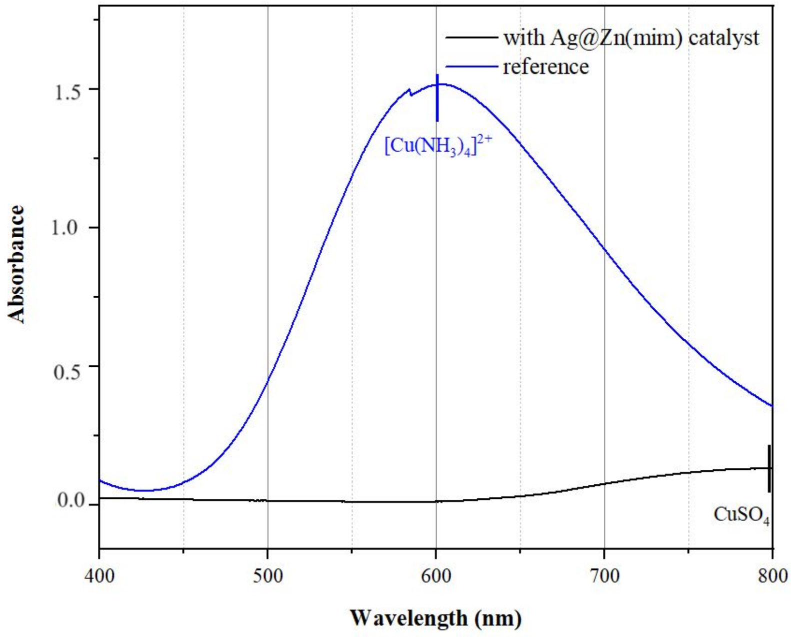

Figure S9 The UV-Vis spectra of the tetra amine copper (II) complex formed in the reference experiment (602 nm, blue line), and of the experiment solution with the catalyst, only the $CuSO_4$ band is observed (above 800 nm, black line).

Copper sulfate undergoes a reaction with ammonium hydroxide, resulting in the formation of a deep blue complex known as tetraamminecopper(II) sulfate. The balanced chemical equation is provided below:

$$CuSO_{4(aq)} + 2NH_4OH \rightarrow Cu(OH)_2 \downarrow + (NH_4)_2SO_4$$

light blue colour

$$Cu(OH)_{2(s)} + 2NH_4OH + (NH_4)_2SO_4 \rightarrow [Cu(NH_3)_4]SO_{4(aq)} + 4H_2O$$

intense blue colour

Crystallite Size Calculation

The modified Scherrer formula used to calculate the mean crystallite size (D_{Sch}) in crystalline materials from X-ray diffraction (XRD) data. The formula takes into account the instrumental correction, and is given by:

$$D_{Sch} = \frac{K\lambda}{\left[(\beta_{obs})^2 - (\beta_p)^2\right]^{1/2} \cos(\phi)}$$

Where:

- D_{Sch} is the average crystallite size.
- K is the Scherrer constant, which depends on the shape of the crystallites (usually assumed to be 0.9).
- λ is the wavelength of the X-ray radiation used.
- β_{obs} is the observed diffraction line broadening (in radians).
- β_p is the instrumental diffraction line broadening (in radians).

- ϕ is the diffraction angle (Bragg) corresponding to the diffraction line used in the analysis.

This correction is important when the broadening of the diffraction lines has contributions from both the crystallite size and the instrument used [3], [4], [5], [6].

Table S4 Turnover frequency (TOF), hydrogen generation rate (HGR), and hydrogen yield in the hydrolysis of NH_3BH_3 catalyzed by AgNPs@Zn(mim) with different concentrations of NaOH at a temperature of 40±0.2 °C.

**NaOH**	**TOF** mol H_2.mol_{cat}^{-1}. min^{-1}	**HGR** mL.min^{-1}. g_{cat}^{-1}	**Yield** (%)	**Time** (min)	**Volume** (mL)
without	4.10	933.51	35.62	34.70	17
1M	3.55	809.26	41.91	47.63	20
2M	4.63	1054.84	48.18	41.14	23
3M	4.93	1124.16	52.38	41.96	25
4M	6.34	1445.82	54.48	33.93	26

Table S5 Crystallite size of AuNPs@Zn(mim) and AgNPs@Zn(mim) catalysts before and after adding NaOH and after five cycles of hydrolysis of NH_3BH_3.

**Sample**	**Phase**	**Crystallite size (nm)**
Zn(mim)	*dia (Zn)*	104.81
AuNPs@Zn(mim)	*dia (Zn)*	104.81
AgNPs@Zn(mim)	*dia (Zn)*	48.88
**PC-** AgNPs@Zn(mim) **– 1M NaOH**	*dia (Zn)*	30.36
**PC-** AgNPs@Zn(mim) **– 2M NaOH**	*dia (Zn)*	36.69
**PC-** AgNPs@Zn(mim) **– 3M NaOH**	*dia (Zn)*	38.44
**PC-** AgNPs@Zn(mim) **– 4M NaOH**	*dia (Zn)*	37.93
PC- AuNPs@Zn(mim) – 5 cycles	*dia (Zn)*	41.41
PC- AgNPs@Zn(mim) – 5 cycles	*dia (Zn)*	41.89

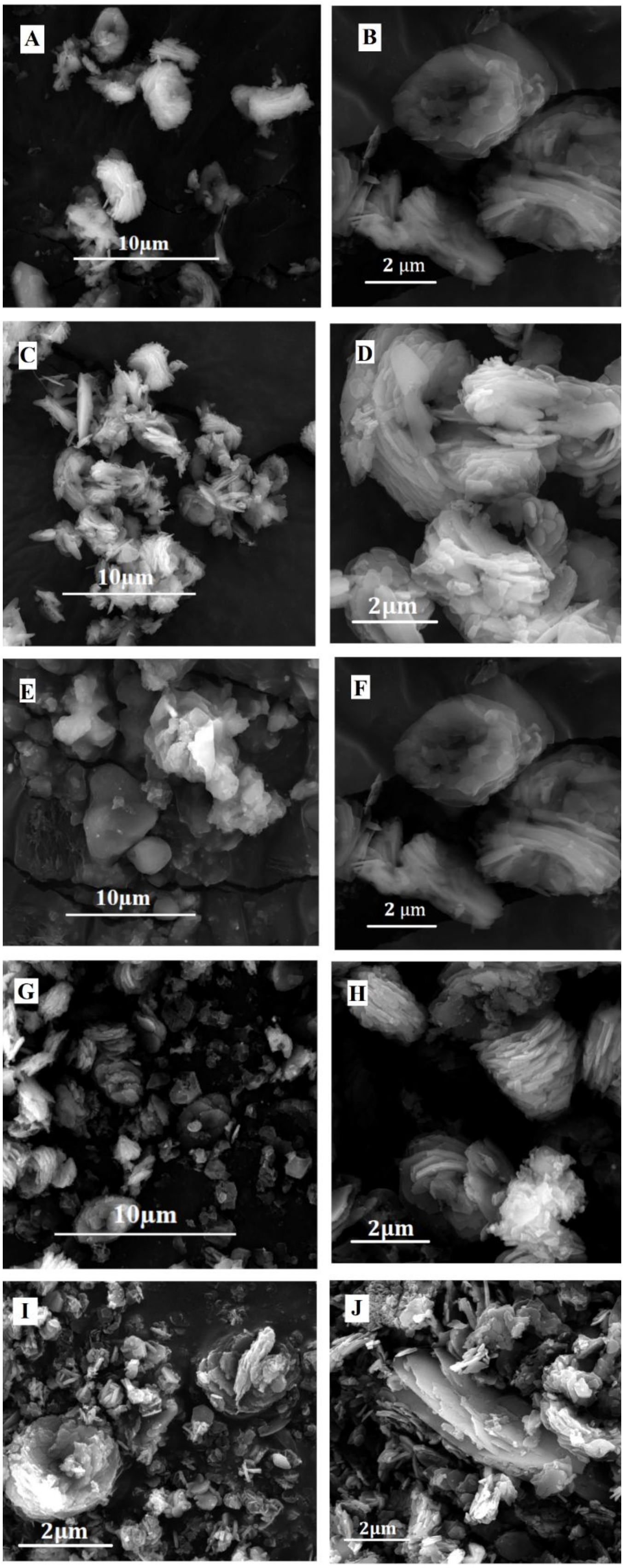

Figure S10 SEM micrographs of the catalysts after hydrolysis of NH_3BH_3 at a temperature of 40±0.2 °C. PC-AgNPs@Zn(mim) sample after basic catalysis: A-B) 1M NaOH, C-D) 2M NaOH, E-F) 3M NaOH and G-H) 4M NaOH. Sample after 5 catalytic cycles: I) PC-AuNPs@Zn(mim) and J) PC-AgNPs@Zn(mim).

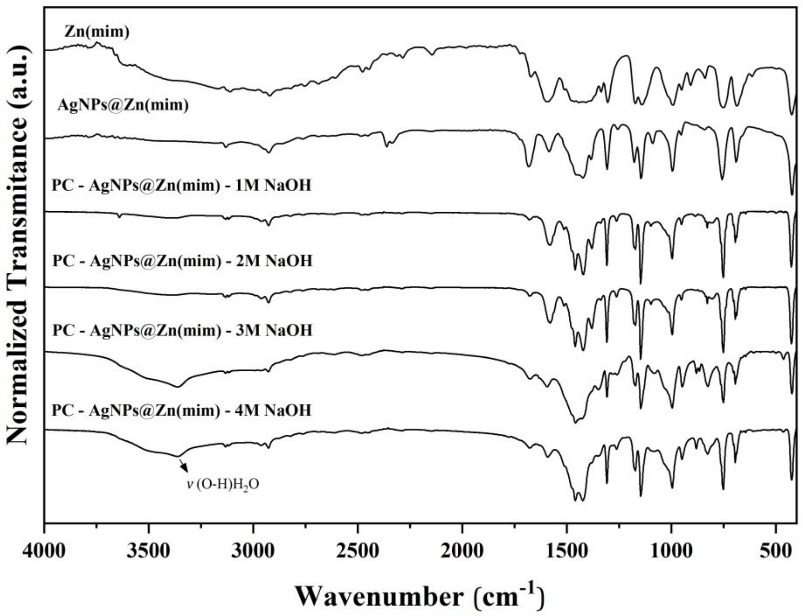

Figure S11 FTIR spectra of the post-catalysis AgNPs@Zn(mim) isolated from NH_3BH_3 hydrolysis carried out with different NaOH concentrations (1-4 M).

Table S6 - Turnover frequency (TOF), hydrogen generation rate (HGR), and hydrogen yield for AuNPs@Zn(mim) and AgNPs@Zn(mim) catalysts in NH_3BH_3 hydrolysis for 5 cycles at a temperature of 40±0.2 °C.

Cycle	TOF<br>mol $H_2.mol_{cat}^{-1}. min^{-1}$	HGR<br>$mL.min^{-1}. g_{cat}^{-1}$	Yield<br>(%)	Time<br>(min)	Volume<br>(mL)
		**AuNPs@Zn(mim)**			
1	8.25	1030.75	29.34	30.66	14
2	7.10	886.84	27.24	33.09	13
3	6.23	778.81	27.24	37.68	13
4	5.22	651.90	23.05	38.09	11
5	4.80	599.40	20.95	37.66	10
		**AgNPs@Zn(mim)**			
1	4.36	995.15	35.62	32.25	17
2	3.78	862.04	35.62	37.23	17
3	3.67	837.30	35.62	38.33	17
4	3.58	817.05	35.62	39.28	17
5	3.05	695.94	31.43	40.69	15

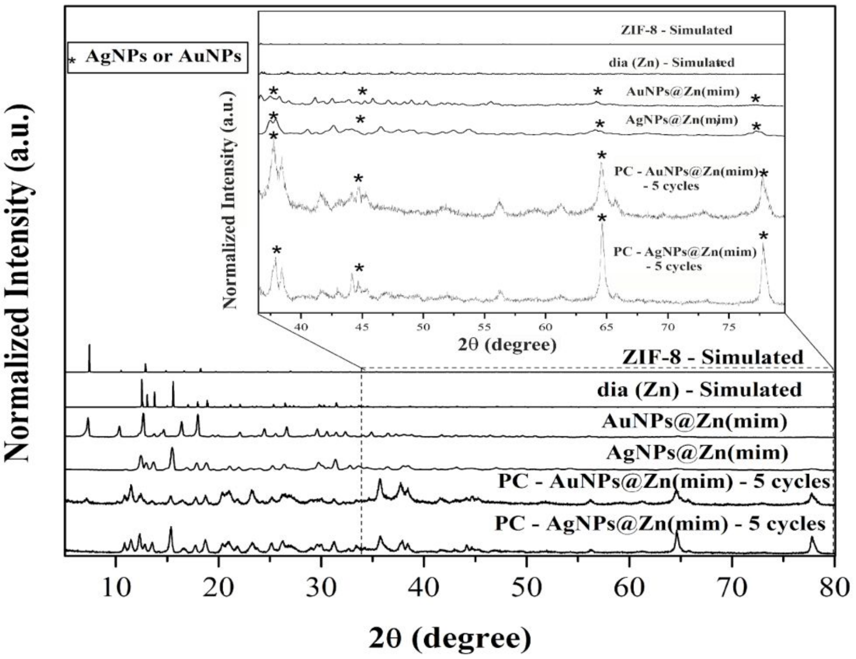

Figure S12 Diffractograms of the catalysts – AuNPs@Zn(mim) and AgNPs@Zn(mim) after five cycles of NH_3BH_3 hydrolysis.

Synthesis of metallic nanoparticles by the chemical reduction method

Gold nanoparticles (NPsAu) with reducing agent sodium citrate: The synthesis of NPsAu was carried out with some concentration modifications based on the chemical reduction proposed by Turkevich in 1951, using trisodium citrate as the reducing agent [1]. The procedure is based on the following reaction:

$$2HAuCl_4 + 3C_6H_8O_7Na_3 + 3H_2O \rightarrow 2Au^0 + 3C_6H_8O_7 + 3Na^+ + 8HCl + 1/2\,O_2$$

In a beaker with 30 mL of boiling chloroauric acid ($HAuCl_4.3H_2O$) [0.5 mmol L^{-1}] under stirring, 20 mL of sodium citrate ($Na_3C_6H_5O_7.2H_2O$) [34 mmol L^{-1}] were added. Then, the boiling mixture was stirred for 6 minutes. During this time, the pink color of the solution appeared, a characteristic color of gold nanoparticles. The obtained nanoparticles were analyzed by UV-visible spectroscopy (Fig. S13).

Silver nanoparticles (NPsAg) with reducing agent sodium borohydride: The synthesis of NPsAg was carried out through the chemical reduction of $AgNO_3$ with $NaBH_4$, a variation of Turkevich's method from 1951 [7], [8], [9]. The chemical reduction reaction of Ag^+ with $NaBH_4$ is given by the equation below.

$$AgNO_3 + NaBH_4 \rightarrow Ag^0 + 1/2H_2 + 1/2B_2H_6 + NaNO_3$$

In an Erlenmeyer flask with 50 mL of sodium borohydride solution ($NaBH_4$) (2 mmol/L)], under the previous cooling in an ice bath for about 20 minutes, magnetic stirring was started. Silver nitrate solution ($AgNO_3$) (3.2 mL, 1 mmol/L) was added drop by drop into the solution of $NaBH_4$ until a golden yellow colloidal solution formed. Next, the solution was stirred in an ice bath for 40 minutes to stabilize the system and then stored. Silver nanoparticles were analyzed by UV-Visible Spectroscopy (Fig. S13).

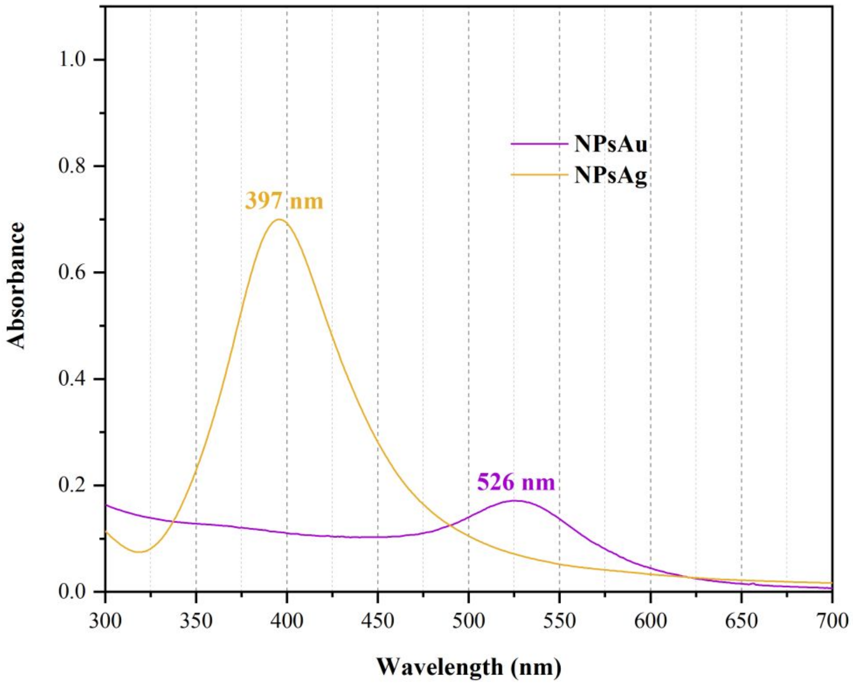

Figure S13 The UV-Vis spectra of synthesized gold (NPsAu) and silver (NPsAg) nanoparticle colloidal solutions.

References

[1] O. Karagiaridi, M. B. Lalonde, W. Bury, A. A. Sarjeant, O. K. Farha, and J. T. Hupp, "Opening ZIF-8: A catalytically active zeolitic imidazolate framework of sodalite topology with unsubstituted linkers," *J Am Chem Soc*, vol. 134, no. 45, pp. 18790–18796, Nov. 2012, doi: 10.1021/ja308786r.

[2] Q. Shi, Z. Chen, Z. Song, J. Li, and J. Dong, "Synthesis of ZIF-8 and ZIF-67 by steam-assisted conversion and an investigation of their tribological behaviors," *Angewandte Chemie - International Edition*, vol. 50, no. 3, pp. 672–675, Jan. 2011, doi: 10.1002/anie.201004937.

[3] F. T. L. Muniz, M. A. R. Miranda, C. Morilla Dos Santos, and J. M. Sasaki, "The Scherrer

equation and the dynamical theory of X-ray diffraction," *Acta Crystallogr A Found Adv*, vol. 72, no. 3, pp. 385–390, May 2016, doi: 10.1107/S205327331600365X/HTTPS://JOURNALS.IUCR.ORG/SERVICES/RSS.HTML.

[4] A. Valério and S. L. Morelhão, "Usage of Scherrer's formula in X-ray diffraction analysis of size distribution in systems of monocrystalline nanoparticles," Nov. 2019, Accessed: Aug. 27, 2024. [Online]. Available: https://arxiv.org/abs/1911.00701v1

[5] P. Scherrer, "Bestimmung der Größe und der inneren Struktur von Kolloidteilchen mittels Röntgenstrahlen," *Nachrichten von der Gesellschaft der Wissenschaften zu Göttingen, Mathematisch-Physikalische Klasse*, vol. 2, pp. 98–100, 1918.

[6] U. Holzwarth, N. Gibson, "The Scherrer equation versus the 'Debye-Scherrer equation' ", Nature Nanotechnology, 6, 534 (2011)

[7] E. S. SOUZA, "Desenvolvimento de filmes compósitos para aplicação antibacteriana bioinspirados na superfície super-hidrofóbica de plantas," Feb. 2019, Accessed: Jun. 30, 2020. [Online]. Available: https://repositorio.ufpe.br/handle/123456789/35473

[8] T. da S. Borralho, "As aplicações da Prata na Nanotecnologia Farmacêutica. Dissertação/tese.Universidade Lusófona de Humanidades e Tecnologias Escola de Ciências e Tecnologias de Saúde. Lisboa, 2017.," 2017.

[9] J. Turkevich, P. C. Stevenson, and J. Hillier, "A study of the nucleation and growth processes in the synthesis of colloidal gold," 1951. doi: 10.1039/DF9511100055.
